# Valorization of rare earth processing byproducts for agriculture usage

**DOI:** 10.1038/s41598-021-93704-9

**Published:** 2021-07-27

**Authors:** Mohamed Musa Hanafi, Parisa Azizi, Sheu Tijani Akinbola, Roslan Ismail, Abdul Rahim Sahibin, Idris Wan Mohd Razi, Aznan Fazli Ismail

**Affiliations:** 1 Laboratory of Climate-Smart Food Crop Production, Institute of Tropical Agriculture and Food Security, Universiti Putra Malaysia, 43400 Serdang, Selangor Malaysia; 2 Laboratory of Plantation Science and Technology, Institute of Plantation Studies, Universiti Putra Malaysia, 43400 Serdang, Selangor Malaysia; 3 Department of Land Management, Faculty of Agriculture, Universiti Putra Malaysia, 43400 Serdang, Selangor Malaysia; 4 Environmental Science Program, Faculty of Science and Natural Resources, Universiti Malaysia Sabah, 88400 Kota Kinabalu, Sabah Malaysia; 5 Centre for Earth Sciences and Environment, Faculty of Science and Technology, Universiti Kebangsaan Malaysia, 43600 Bangi, Selangor Malaysia; 6 Nuclear Science Program, Faculty of Science and Technology, Universiti Kebangsaan Malaysia, 43600 Bangi, Selangor Malaysia; 7 Centre for Frontier Sciences, Faculty of Science and Technology, Universiti Kebangsaan Malaysia, 43600 Bangi, Selangor Malaysia

**Keywords:** Environmental sciences, Solid Earth sciences

## Abstract

Sandy texture soil, a major problem for agriculture requires structure and capacity improvements. However, utilization of soil conditioner may arrest this problem. This research was carried out to investigate the accumulated levels of metal ions and radionuclides in water, soil and plants following phosphogypsum organic (PG organic) added to a sandy soil for 23-month in 3 cropping seasons. The condition in the field was simulated in the laboratory using an open leaching column for 30-day under constant but different pH of leachant. More ions were released at pH < 4.6 and decreases greatly at pH > 5.6. The metal ions measured in the surface and borehole water, and soils were below the target values for respective standard raw drinking water. The metal ions did not accumulate in soil, plant and grain, and water as indicated by biological accumulation coefficients, contamination factors, I-geo index and pollution load index in a sandy soil that received the PG organic. Naturally occurring radionuclide concentrations, such as ^226^Ra, ^228^Ra, and ^40^K, in soil and plant tissue were found to be lower than the average value reported by several earlier studies. Under field condition the pH of water (i.e., rainfall) was greater than pH 5.6, thus renders PG organic became less soluble. There was no leaching of natural occurring radionuclides to the groundwater. Therefore, the application of PG organic to the studied soil had no impact on the soil, plants, and water and suitable as a soil conditioner in sandy texture soils.

## Introduction

Phosphogypsum (PG) is typically a solid waste and mostly made of gypsum (CaSO_4_.2H_2_O). It may contain further solid segments (e.g., fluorides), traces of unreacted phosphate rock (PR) and organic matter (OM). In addition, minor liquid waste components (remains of process waters) can be included in PG^1^. It is estimated that the total amount of PG produced up to 2006 was approximately 6 billion tons, of which 2.2 billion tons (37%) were manufactured in the United States^2^.

The emergence of different studies focused on the discovery of new recycling alternatives for PG, such as the manufacture of building materials, the application in agriculture, the acquisition of mineral resources or environmental applications, has promoted the attractive economic potential of PG. The PG has commonly been recognized as an amendment to improve the physico-chemical characteristics of soils because of its high content in certain elements, such as calcium (Ca), phosphorus (P) and sulfur (S). This route of valorization is not new; the possible use of this by-product in agriculture was reviewed by Alcordo and Rechcigl^3^. Since then, many studies have been allocated to the agricultural uses of PG^4–9^. Notably the studies have been carried out to improve crop yield and soil structure^10^, decreasing soil erosion^11^, treating soils that are acidic or metal-rich^12,13^ or increasing the available soil concentrations of S and P^14^. The analysis of PG impurities along with their mobility and bioavailability are of crucial significance to ensure a healthy long-term application in agriculture. To simulate the movement of contaminants from PG modified soils to agricultural products, some authors have tackled this problem by adding various extractants to PG, such as by measuring leaching amounts of radionuclides and metals from Brazilian PG in comparison to PR^15^, *aqua regia* digestion and sequential extractions^16,17^.

In this research we used PG organic, which contains neutralization underflow (NUF) residue, water leached purification (WLP) residue from rare-earth elements processing plant, and organic filler material in a ratio of 2:1:7. In order, to develop specific soil quality remediation and having soil with a great preference, which will be important requisites in the development of agricultural product, PG organic has been applied to improve sandy soils. The organic material used as filler is derived from composted oil palm empty fruit bunch (COPEFB), a by-product from palm oil processing mill. This soil contains more than 80% of sand fraction is a problematical soil in Southeastern Asia, including Malaysia and Indonesia^18^. It is unsuitable for agricultural purposes because of its weak structure, deficient of nutrients, low capacity of water retention, high soil temperature, and ultimately inadequate supports of plants to grow^19^. However, it is assumed that application of fertilizer in combination of PG organic may improve sandy soil structure and quality. The organic fraction may play an important role to held nutrients and for the formation of soil structure. Successful utilization of this PG organic in this soil might be used for other problematic soils worldwide. To the best knowledge of the authors, there was no such study relating the usage of PG organic for improvement of sandy soil and its effect on soil, water, and plants ecosystems.

On the other hand, one of the main sources of metal ions in water (surface and ground water), soil and plants is leachate migration^20^. Therefore, the objectives of this study were (i) to observed migration of selected ions related to plant nutrition in the short-term laboratory experiment and (ii) to investigate the accumulated levels of metal ions (MIs) (e.g., As, Cd, Ce, La, Se, Sr, Th, Ra, Zn, B, Mn, Pb, Cr, Ag, Ba and Hg) and natural radioactivity concentrations, such as ^226^Ra, ^228^Ra, ^238^U and ^232^Th in water, soil, and plants between sandy soil under PG organic treatment and control (normal sandy soil) in real field situation.

## Materials and methods

### Phosphogypsum organic

This PG organic was developed by Lynas, the world's second largest producer of rare earth materials and the Universiti Putra Malaysia (UPM) as a ‘soil conditioner’ in 2015. It contains neutralization underflow (NUF) residue, water leached purification (WLP) residue from rare-earth elements processing, and organic filler material. Lynas Malaysia, a subsidiary of Lynas Corporation Ltd (Australia), is one of the largest and most modern rare earths separation plants in the world. At optimum production, Lynas Advance Materials Plant (LAMP) produces WLP and NUF at 64,000 and 124,000 tons/year, respectively. These industrial by-products are an environmental and safety issues not only in Malaysia, but also worldwide^21^. The PG organic (Supplementary characterization data—I) was prepared in the LAMP complex and transported by a special land transport following the guideline approved by the Department of Environment Malaysia for immediate use at the experimental site.

### Soils

**Figure 1:**
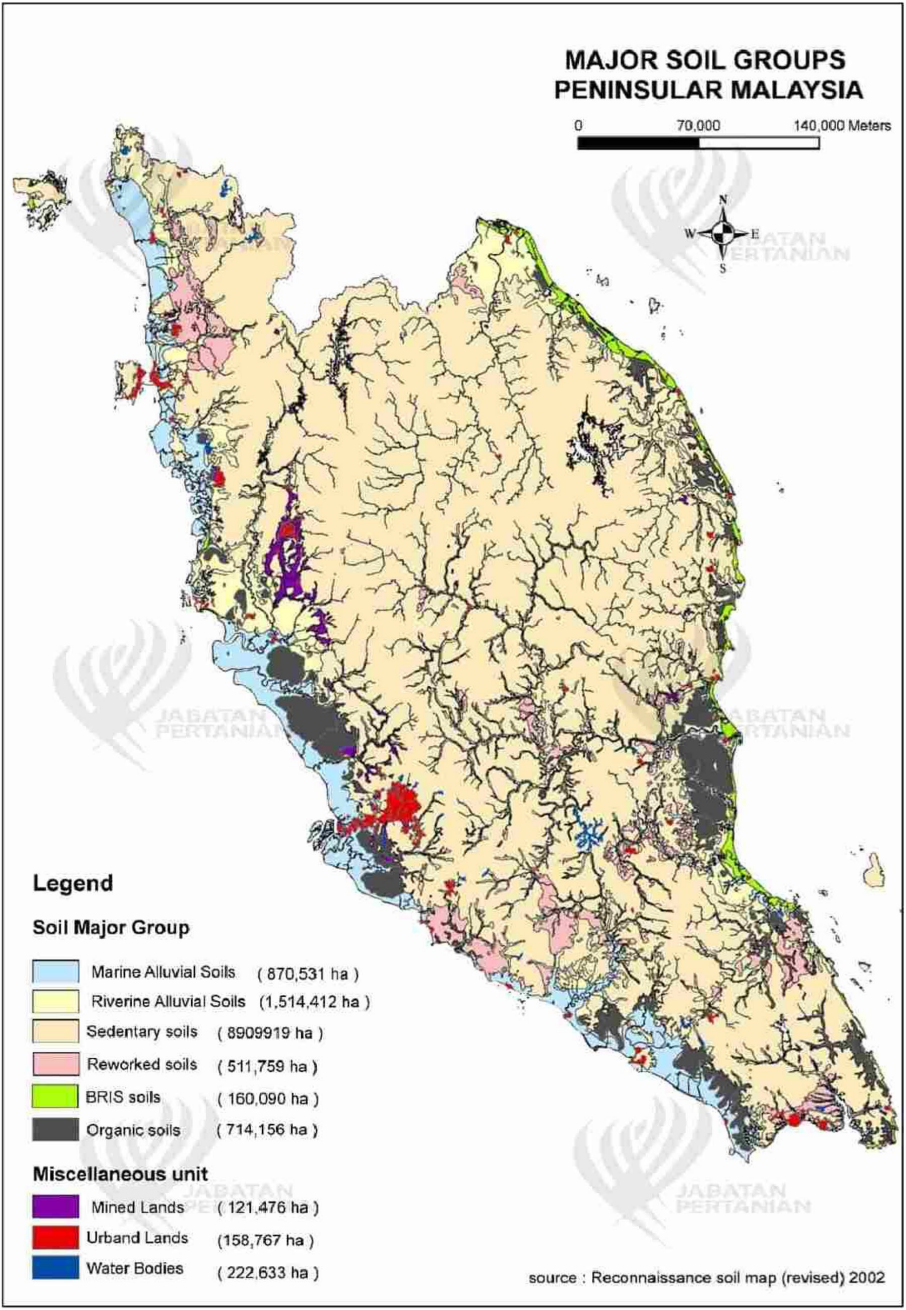
The extent of sandy soils distributions in Peninsular Malaysia^70^.

A group of sandy (> 80% sand fraction) soils developed from marine deposits on a coastline (Fig. 1) and containing a spodic horizon (spodosol) (Typic Haplorthord) or without spodic horizon (Typic Quartzisamment) is a problematical soil in Southeastern Asia including Malaysia and Indonesia^18,19^. These soils are developed on ridges and swales, which is locally known as the beach ridges interspersed with swales (BRIS). During monsoon seasons, the steep cliffs layers erosion and deposition of residues and sand from the sea is developed by moving sea water and create BRIS soil containing coarse sand components^20^. This soil is categorized into seven soil series based on drainage, depth and soil profile, including Rhu Tapai, Rusila, Rudua, Rompin, Merchang, Jambu and Baging^19^.

### Simulation of ions released from PG organic in a sandy soil column

Leaching columns of diameter 5.5 cm were cut to length of 30 cm of poly vinyl chloride (PVC) pipe and then transported to the field. In the field leaching columns were pressed into the soil at a depth of 0–30 cm, cut and then covered at the two ends and transported back to the laboratory for the experimental set up.

**Figure 2:**
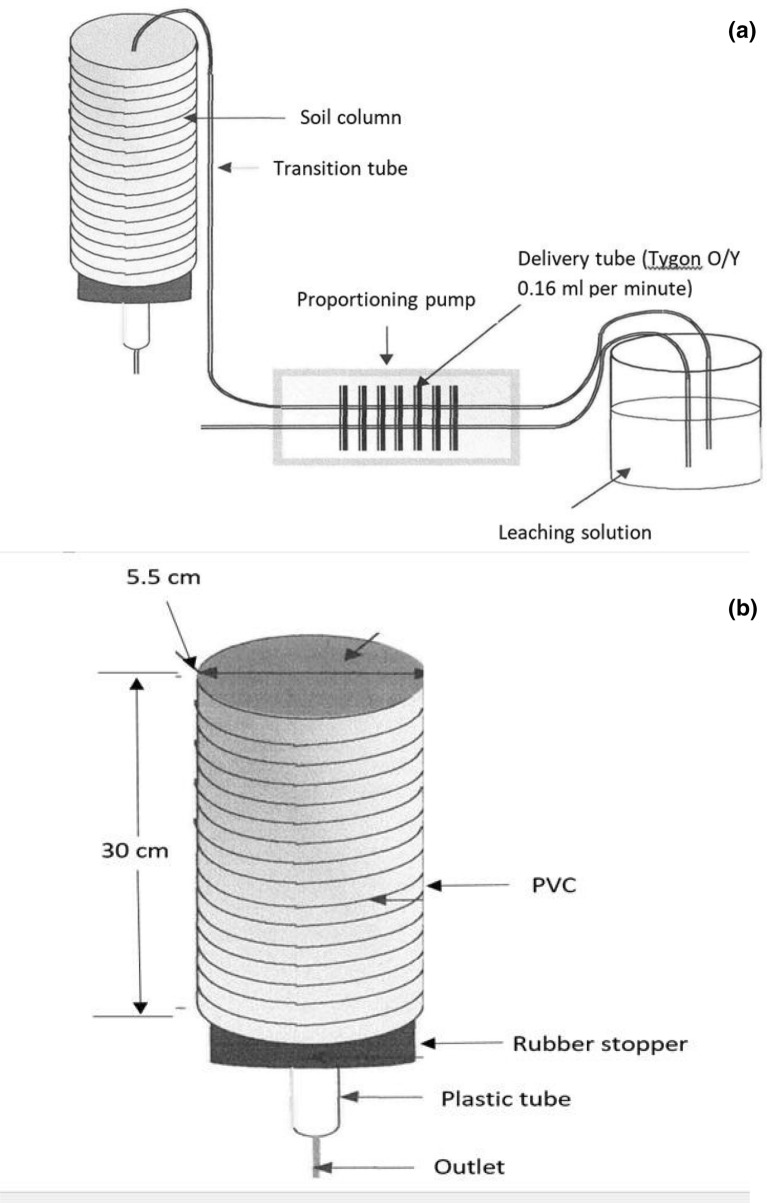
Schematic diagram of the experimental set-up (**a**) and the constructions of soil leaching column (**b**) using a PVC pipe.

In the laboratory, rubber stoppers fitted with tubing outlet were fixed to the bottom of each of the soil column in preparation for leaching experimental setup (Fig. 2). The soil columns were equilibrated on a rack after allowing water to flow up by capillary rise through an outlet at the bottom of the soil columns to a saturated point and then allowed to drain completely. Top 2 cm of the soil in the column was removed and PG organic was mixed thoroughly in top of soil of the column at the rate of 28 tons/ha (equivalent to 14.0 g per column) (Fig. 2a). Then, the leaching solutions, varied in acidities (pH 1–9) were delivered using a proportioning pump with tubing (Tygon Y/Y) of flow rate of 0.16 mL per minute, and then allowed to drop on a filter paper placed on the soil surface in the column (Fig. 2b). This procedure was successfully used to measure the dissolution of PR in acid Malaysian soils in an open-leaching column^22^. The top of the columns was covered by transparent waterproof tape. Leaching of sandy soil amended PG organic continued up to 30 days, which was approximately equivalent to the total rainfall for 1 year (2,400 mm) in the region.

Leaching solution of five different pH values was prepared and stored in a plastic container, which was used for a period of 30 days. The leaching solution was prepared according to Pearse^23^; pH 1.2 solution was prepared with 0.2 M HCl and 0.2 M KCl, while 0.1 M glacial acetic acid and 0.1 M Na acetate was used to prepare leaching solution of pH 3.6, 4.6 and 5.6, and finally pH 8.8 solution was prepared with 0.2 M glycine and 0.2 M NaOH.

Leachate was collected for every 3 days, sampled, and kept in the refrigerator for the period of 1 month of experiment. The pH and electrical conductivity (dS/m) reading of the leachate were taken using a pH meter (Model TRANS BP 3001) and EC meter (Model TRANS BC 3020), respectively. Leaching solution was analyzed using inductively coupled plasma mass spectrometry (ICP-MS) (Model: Perkin Elmers Elan 9000, Shelton, USA) for the following ions, such as phosphorus (P), calcium (Ca), magnesium (Mg), cupper (Cu), iron (Fe), manganese (Mn) and zinc (Zn); while aluminum (Al), strontium (Sr), chromium (Cr), thorium (Th) and lead (Pb).

At the end of the experiment (30 days), the soil columns were allowed to drain completely and were cut into 5 cm each, and the soil was air-dried, extracted with Mehlich 3 extractant^24^ and analyzed for the same nutrients listed above using AAS analyzer and ICP-MS, respectively.

### Field experimental site

**Figure 3:**
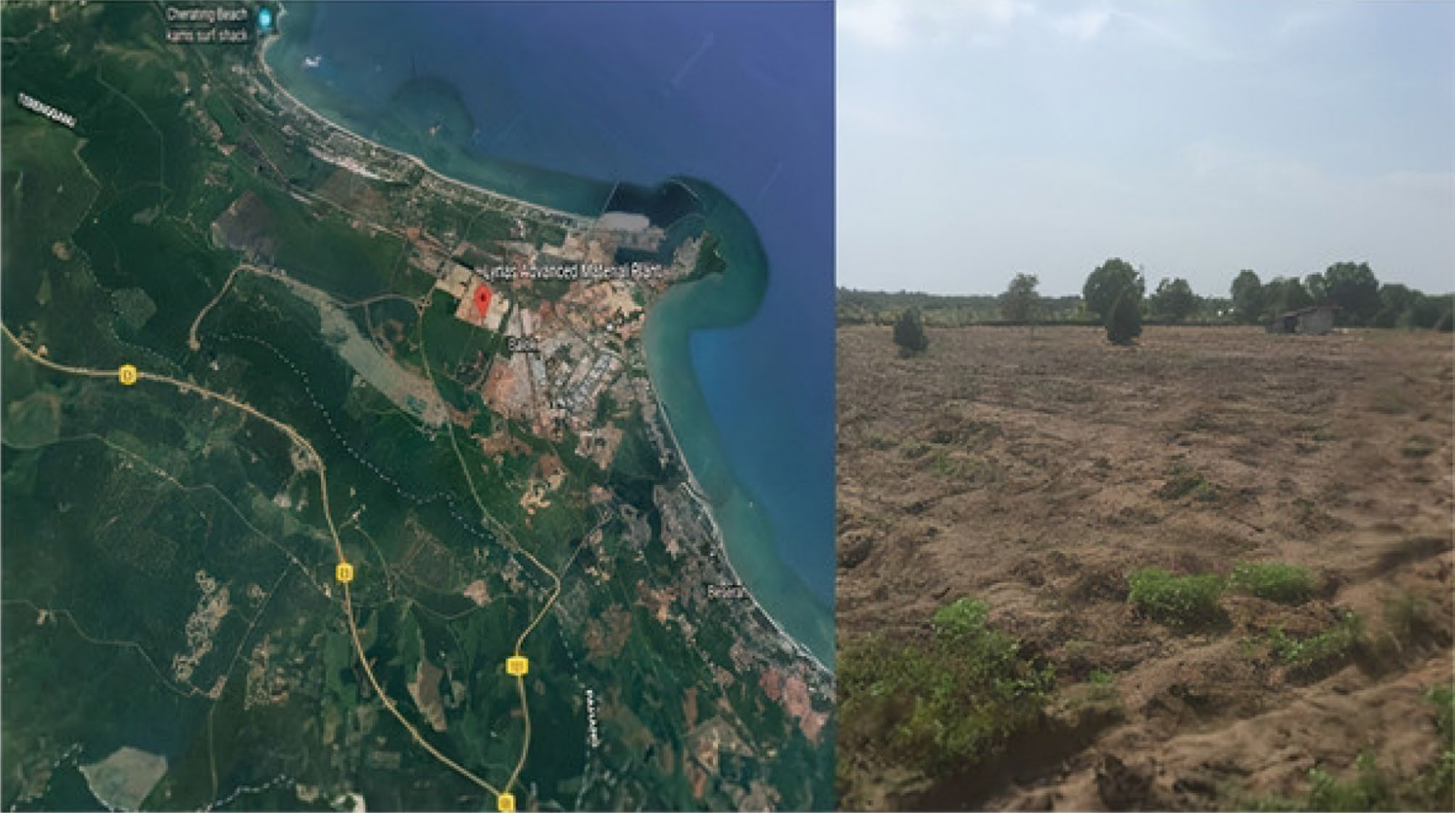
The location of the experimental site and the Lynas Advance Material Plant (LAMP)^71^; Note: Scale of map; 1.0 cm = 1.0 km.

The field trial was conducted on 1.62 ha at Kampung Darat Sungai Ular, Pahang (GPS 3°50′51"N, 103°21′46"E) as shown in Fig. 3.

The implication of PG organic application to the environment was monitored for three planting seasons (23-month) beginning in 2015 with 4-month cycles for each season. The residual effect of the PG organic conditioner was measured for three test crops, including corn (*Zea mays* L.), kenaf (*Hibiscus cannabinus* L.) and guinea grass (*Panicum maximum*).

### Plot design and treatment

**Figure 4:**
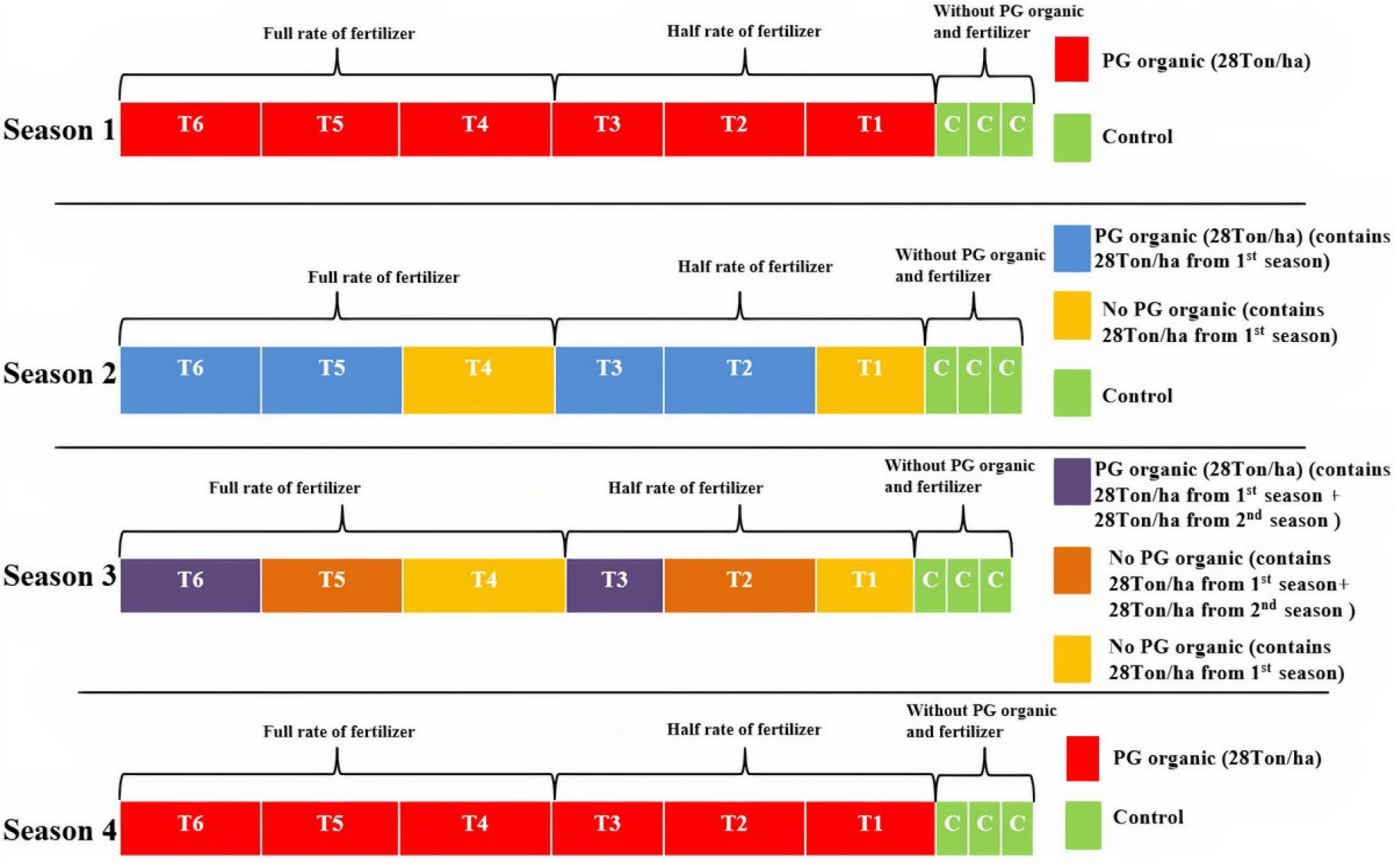
The sub-plots receiving the PG organic conditioner treatment for 3 cropping seasons for kenaf, maize, and guinea grass. Blue represents the sub-plot receiving the soil conditioner for each cropping season, and yellow represents the sub-plot receiving residual treatment.

The randomized complete block design (RCBD) was applied on the experimental site with three replications for two seasons. There are seven treatments applied as shown in Fig. 4.

The PG organic was applied at the top of soil surface and mixed thoroughly in the top 15 cm soil depth using a rotovator machine. There was allowed to equilibrate for 7 days before cropping of plants. Fertilizer treatments based on crop recommended rate and frequencies of application were added to top of the soil under the plant canopy, when permissible based on soil and foliar analyses. A drip irrigation water system was applied to each plot using black plastic tape tubing. The water was supplied when necessary to achieve the target moisture content of soil field capacity (FC). All soil, plants and water characteristics were compared between control plots (without PG organic and fertilizer) and those plots received maximum amount of PG organic as conditioner (T6).

### Sampling of soils, water and plants

Soils were sampled on three occasions: before the PG organic application (base line), after the PG organic application (one month after application) and at harvest. Soil samples, water samples and plant samples were taken for analysis in the three seasons of field experiment. Every season the soil and water samples were taken before PG organic applications, one month after PG organic application and at harvest. Plant samples were taken during harvest. In total 18 composite soil samples corresponding to the treatment were taken whereas three soil samples were for control every season. Water samples were collected from five boreholes available within the study area and two surface water samples from the nearby river every time during sampling.

Topsoil (0–30 cm) samples were taken randomly in a composite manner from the designated plot to determine their physico-chemical properties, plant nutrients, metal ions and natural radionuclides contents. Approximately 2 kg of composite topsoil (0–30 cm) samples was collected and kept in a plastic bag. Meanwhile, water was sampled from boreholes within or near the plots, and surface water was sampled in the nearby Sungai Ular. In situ water parameters, such as pH, salinity, dissolved oxygen, electrical conductivity, temperature, and total dissolved solids were examined in the field using an YSI 556 MPS handheld multi-parameter water quality meter. All water samples collected for laboratory analysis were kept in an ice box with a temperature below 4 °C and contained the addition of several drops of HNO_3_ acid to control the pH below 2. The aboveground mass of plants was cut and sampled for their stems, leaves and grain for corn. The summarized of detailed information regarding the samples is shown in Table 1.

**Table 1:** Locations of soil, plant, and water sampling at the experimental plot.

Season	Sample type	Sample ID	Note
2nd Season	Soil	Kenaf-soil-control	Control sample without PG organic
		Kenaf-soil-control	
		Kenaf-soil-PG organic	Soil treated with PG organic
		Guinea grass-soil-PG organic	
	Water	BH-1	Underground water
		BH-2	
		BH-3	
		BH-4	
		BH-5	
		SW-1	Surface water (river)
		SW-2	
	Plant	Kenaf-stem-Control	Plant growing without PG organic
		Guinea grass-leaf-Control	
		Kenaf-stem-PG organic	Plant Growing with PG organic
		Guinea grass-leaf-PG organic	
3nd Season	Soil	Kenaf-soil-control	Control sample without PG organic
		Kenaf-soil-control	
		Kenaf-soil-PG organic	Soil treated with PG organic
		Guinea grass-soil-PG organic	
	Water	BH-1	Underground water
		BH-2	
		BH-3	
		BH-4	
		BH-5	
		SW-1	Surface water (river)
		SW-2	
	Plant	Kenaf-stem-control	Plant growing without PG organic
		Guinea grass-leaf-control	
		Kenaf-stem-PG organic	Plant Growing with PG organic
		Guinea grass-leaf-PG organic	

### Characterization of soil properties

Soils were tested for their particle size distribution and organic matter content. The particle size distribution was determined using the pipette method together with dry sieving^25^, whereas organic matter was determined by the loss on ignition method^25^. Soil chemical tests involved the pH (H_2_O), plant nutrient contents (Ca, Mg, K, and P), organic carbon content, electrical conductivity, and metal ions. The soil pH in water was determined at soil: solution ratio (1/2.5) and measured using a pH meter (Model: Metrohm 827, Riverview, FL, USA). Organic carbon was measured with the Walkley and Black method^26^. Electrical conductivity was measured in saturated gypsum extracts^27^ (Model: Mettler Toledo SevenEasyTM Conductivity Meter S30, Hamilton, New Zealand). The total C, N, and S were measured using a CHNS analyzer (Model: LECO CHNS-932).

## Analysis of Metal Ions

### Soils

Soil samples were dried at room temperature, ground, and sieved to pass through a 2-mm sieve. For the determination of metal ion contents, the soil was pulverized to pass through a 63-micron sieve. Plant samples were cleaned in running water and rinsed with deionized water at least three times before being cut into small pieces, dried and ground using a micro-hammer mill (Model: IKA mills MF 10.1, IKA Works GmbH & Co., Staufen, Germany). The total metal ion contents in the soil was extracted with nitric acid and perchloric acid in a 3:1 ratio^28^. Metal ion contents (As, Cd, Ce, La, Se, Sr, Th, Zn, B, Mn, Pb, Cr, Ag, Ba and Hg) were extracted by the wet digestion method. Metal ions in solution were determined using the ICP-MS (Model: Perkin Elmers Elan 9000, Shelton, USA).

### Water

The water analysis followed the standard methods proposed by American Public Health Association, APHA^29^. Metal ion contents in water (e.g., As, Cd, Ce, La, Se, Sr, Th, Ra, Zn, B, Mn, Pb, Cr, Ag, Ba, Al, and Hg) were measured using ICP-MS (Model: Perkin Elmers Elan 9000, Shelton, USA).

### Plants

The plant parts were separated into roots, stems, leaves and grain and analyzed for metal ions and plant nutrient. The total metal ion contents of the plants were extracted with nitric acid and hydrogen peroxide in a 3:1 ratio^30^. The metal ion contents determined included As, Cd, Ce, La, Se, Sr, Th, Zn, B, Mn, Pb, Cr, Ag, Ba, Al, and Hg. Metal ions in solution extracts were determined using ICP-MS (Model: Perkin Elmers Elan 9000, Shelton, USA).

## Natural radioactivity analysis

Sample preparation-Each sample was prepared accordance to the International Atomic Energy Agency (IAEA) guidelines^31,32^. The soil and plant samples were cleaned and dried in the oven at 105 °C for 72 h, until a constant weight was achieved. Soil and plant samples were then left to cool at room temperature, ground using a grinder and sieved with a 500 µm sieve size to ensure the homogeneity of the samples. Meanwhile, water samples were filtered out from impurities using a clean dried sieve cloth. The samples were then packed into Marinelli counting beaker, sealed and for 30 days to attain secular equilibrium between the parents’ radionuclides (^238^U and ^232^Th) and their respective progenies.

### Gamma spectrometry analysis

Natural radioactivity in samples was determined after 12 h of counting using a gamma spectrometry system with a Canberra High-purity Germanium Detector (HPGe) coupled to a multichannel analyzer. The HPGe detector was shielded inside CANBERRA model-747 lead shielding coated with tin and copper to provide a low background environment. The radioactivity concentrations of ^226^Ra, ^232^Th and ^40^K were determined through gamma-ray photo peaks of 1764 keV (^214^Bi), 2614 keV (^208^Tl) and 1460 keV (^40^K) with the corresponding emission probability of 15.2, 99.8 and 10.7%, respectively. The gamma spectrometry system was calibrated using a standard source with a mixture of ^22^Na, ^51^Cr, ^57^Co, ^60^Co, ^85^Sr, ^88^Y, ^109^Cd, ^113^Sn, ^137^Cs, ^123^Te and ^241^Am radionuclides. The analysis of gamma spectra was performed using Genie-2000 software supplied by CANBERRA. The IAEA Soil-375 (certified reference material), IARMA-004 (certified reference material) and 0802 Silica Standard (Multinuclide Standard Mixed with Silica) were used as reference in this research. The counting errors were quoted at 1-sigma absolute. Natural radionuclide concentrations in soil and plant samples were determined using Eqs. (1) and (2), respectively, as follows^33,34^.

1 $${\text{W}}_{{\text{s}}} = \frac{{M_{{rm}} \times A_{s} }}{{M_{s} \times A_{{rm}} }} \times {\text{ W}}_{{{\text{rm}}}}$$

2 $${\text{W}}_{{\text{s}}} = \frac{{A_{s} }}{{\varepsilon _{\theta } M_{s} }}$$

The W_s_ and W_rm_ are radionuclide concentrations for sample and reference materials in Bq/kg, M_s_ and M_rm_ are sample and reference material masses (g), while, As and Arm are the activities (net count per second, cps) for sample and reference materials, respectively. The Є and θ are the energy efficiency and abundance of the interest gamma energy peak, respectively. The accuracy of the procedure was checked by measuring the IAEA Soil-375 the lower limit detection (LLD) and the measured activity of ^226^Ra, ^232^Th and ^40^K under the same condition and samples (Supplementary data available -II).

## Pollution indices related to metal ions and radionuclides

The guidelines used as a comparison to the metal ion contents in water were the available standard, such as the new Dutch List^35^, Malaysian Guidelines for Raw Water Quality Criteria^36^ and the National Water Quality Standard (NWQS)^37^. The metal ion contents in soil were compared with the new Dutch List^35^, and the metal ion contents in the grain were compared with values from the Malaysian Food Act^38^ and Food Regulation^39^ standard.

### Biological accumulation coefficient

The biological accumulation coefficient (BAC), which is an indicator of the ability of a plant to accumulate a specific metal ion in contrast to the concentration of the metal ion in the soil substrate^40^, was calculated as follows:

3 $${\text{BAC }} = \frac{{{\text{Metal}}\;{\text{concentration}}\;{\text{in}}\;{\text{plant}}}}{{{\text{Metal}}\;{\text{concentration}}\;{\text{in}}\;{\text{soil}}}}$$

where, the metal ion concentrations in the edible parts of the plant and the soil are represented by metal ion concentration in the plant and metal ion concentration in the soil, respectively.

### Geo-accumulation index

Established by Muller^41^, the geo-accumulation index (I-geo) was used to discover how much metal ion has built up in sediments and has been employed in a number of applications and for a number of research purposes. The I-geo is mathematically expressed as:

4 $${\text{I}} - {\text{geo }} = {\text{ log}}_{{\text{2}}} \left( {{\text{Cn}}/{\text{1}}.{\text{5Bn}}} \right),$$

where, Cn = Concentration of element in the soil sample, and Bn = the geochemical background value. Factor 1.5 is used in the equation to take into consideration the chance of disparities in background data because of lithogenic factors. The I-geo scale is made up of seven levels (0–6), from no contamination to heavy pollution.

### Pollution load index

The pollution index (PLI) is composed of contamination factors (CF), which are the quotients given by the division of the concentration of each metal ion. The CF is made of four levels (0–3, low CF to high CF)^42^. The PLI of the site is estimated by gathering the n-root from the CFs, which were found from the metal ions the PLI gathered from each location^43^. The PLI was created by Tomlinson et al.^44^ using the formula below:

5 $${\text{CF}} = {\text{C}}\;{\text{metal}}/{\text{C}}\;{\text{ background }}\;{\text{value}}.$$

6 $${\text{PLI }} = \sqrt[{\text{n}}]{{({\text{CFI}} \times {\text{CF}}2 \times {\text{CF}}3 \ldots \times {\text{CFn}}}}$$

where, CF = contamination factor, n = number of metals, C metal = the metal concentration in soil samples and C Background value = the background value of that metal.

### Determination of radiological hazard index

A radium equivalent (Ra_eq_) was used to estimate radiological hazard index (RHI). The significance of ^226^Ra, ^232^Th, and ^40^K concentrations, with respect to radiation exposure, was defined in terms of Ra_eq_ activity in Bq/kg. The Ra_eq_ was calculated using the following equation^44–46^:

7 $${\text{Ra}}_{{{\text{eq}}}} = {\text{ CRa }} + {\text{ 1}}.{\text{47CTh }} + {\text{ }}0.0{\text{77CK}}$$

where, CRa, CTh, and CK are the activity concentrations of ^226^Ra, ^228^Ra, and ^40^K in Bq/kg, respectively. This equation is based on the estimation that 10 pCi/g of ^226^Ra, 7 pCi/g of ^232^Th and 130 pCi/g ^40^K will produce the same gamma dose rates^45,47^. The value of Ra_eq_ should be less than 370 Bq/kg to be safe for agricultural applications or building materials, as recommended by Nuclear Energy Agency (NEA) and Organization for Economic Co-operation and Development (OECD)^48^.

### Determination of radionuclide uptakes by plant

The uptake of ^226^Ra, ^228^Ra, ^238^U, ^232^Th and ^40^K from soil to plants was determined using a parameter known as the transfer factor (TF), as follows^49^:

8 $${\text{TF }} = ~\frac{{{\text{Cp}}}}{{{\text{Cs}}}}$$

where, C_p_ and C_s_ are the concentrations of radionuclides (Bq/kg) of interest in plants and soil, respectively. The TF is used to describe the soil-to-plant transfer of radionuclides through the plant roots.

### Quality assurance of metal ions and radioactivity analyses

Quality assurance/quality control (QA/QC) was considered using blanks and replicates for the water, soil, and plant tissue samples. The method for water sample was validated by analyzing the standard reference material (SRM) NIST 1640a (Trace Elements in Natural Water), purchased from National Institute of Standards and Technology (NIST)^50^. The method for soil was standardized using procedure of SRM-2711a Montana II soil^51^. In addition, metal ions analysis in plant samples was validated by the SRM 1568b rice flour^52^ and CRM-LGC7162 strawberry leaf^53^.

Furthermore, internal reference materials were used for precision, quality assurance and control (QA/QC) for selected metal measurements. The average values of three replicates were taken for each determination. The precision of analytical procedures was expressed as the relative standard deviation (RSD), which ranged from 5 to 15% and was calculated from the standard deviation divided by the mean. The recovery rates of the studied metals were within 85 ± 15%.

The measured radioactivity concentrations for ^226^Ra (^238^U series), ^228^Ra (^232^Th series) and ^40^K for the gamma spectrometry system were in the range of the 95% confidence level, as proposed by the IAEA for reference material CRM-375 (Supplementary data available—II). Moreover, the MDA analysis indicated that the lower limits of detection for ^226^R, ^228^Ra and ^40^K were 0.7, 0.2 and 1.2 Bq/kg, respectively. Based on both analyses, it can be judged that the gamma spectrometry system was in good condition, and the results were acceptable during the analysis period.

Chemicals, stock solutions, and reagents were obtained from Sigma/Fluka/Merck and were of analytical grade. All glassware was washed with distilled water, soaked in nitric acid (30%) overnight, rinsed in deionized water and air dried before use.

### Statistical analysis

All the data were analyzed using the Statistical Analysis System (SAS) version 9.4 software. All data were subjected to an analysis of variance (ANOVA) statistical analysis to detect the variation of metal ion contents at different sampling times, which coincided with the PG organic application. A comparison of means was performed using the least significant difference (LSD) test for the test crops.

## Results

### Dissolution of PG organic as affected by acidity of leachant

**Figure 5:**
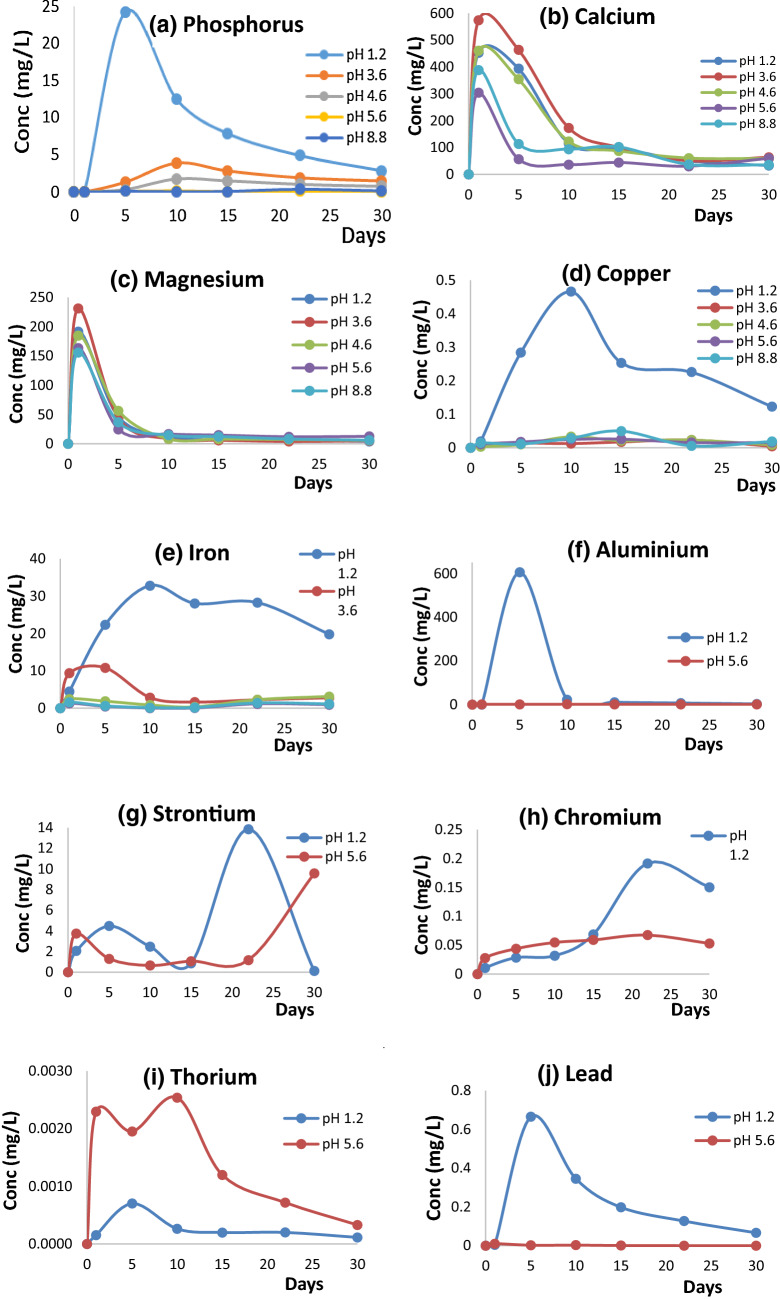
The dissolution of PG organic over the period of 1 month in amended sandy soil column.

Most of the ions in the PG organic dissolved and leached in higher concentration in the earlier days, and gradually reduced by days, irrespective of the pH of leachant (Fig. 3). Each ion leached from soil column differed in quantity to achieve the maximum and minimum concentration. The highest dissolution of phosphate was found in strongly acidic leachant pH of 1.2 but decreased over time (Fig. 5a). The highest concentration (24.3 mg/L) recorded was on day 5, and it decreased to 2.85 mg/L after 25 days.

For Ca and Mg, the highest concentration was found on day 1 with 573.9 and 231.1 mg/L, respectively, as dissolved by pH 3.6 leachant. This value decreased by days and at day 30, the concentration recorded became 63.76 and 4.91 mg/L, respectively (Fig. 5b). For Cu and Fe ions, there were an increased leaching of these elements in the strongly acidic leachant. For both elements, concentration increased by days, up to day 10 in leaching solution of pH 1.2 and later decreased gradually and as low as P, Ca and Mg, both was still able to release more even at day 30 (Figs. 5d and e).

In case for other metal ions, the pH of leachant used was reduced from five to two in this study because there was limited dissolution of ions in most of the leachant pHs, except in the case of pH 1.2, which most elements dissolved in it. The Al, Sr, Cr and Pb were observed to dissolve more in strongly acidic leachant of pH 1.2 than at pH 5.6 (Fig. 5), which is considered as the pH of this soil. The concentration of Al, Sr, Cr and Pb was higher in pH 1.2, except in the case of Th that was dissolved more in the leachant of pH 5.6, though in a very small amount. At pH 1.2, the concentration of Al was higher at the earlier days, and decreased by days, and up to zero at the end of 30 days. The highest concentration was observed on day 5 with 606.1 mg/L and then reduced to 22.0 mg/L in day 10, and finally to 2.5 mg/L in day 30 (Fig. 5f). For other metal ions Sr, Cr, Th and Pb, the highest concentration was observed on day 22, 30, 10 and 5, respectively in lower amount except for Sr at 13.9 mg/L. Among all, only Th was dissolved in pH 5.6 leachant in a higher amount than at pH 1.2, with just 0.0025 mg/L as the highest concentration throughout the study period. The highest cumulative quantity of dissolved PG organic at the respective of pH of leachant in the amended sandy soil column at the end of 30 days (in mg) was 658.4 (Al), 29.0 (Sr), 0.40 (Cr), 0.007 (Th), and 1.5 (Pb) (Supplementary data available—III).

### The pattern of ions movement in the soil column

**Figure 6:**
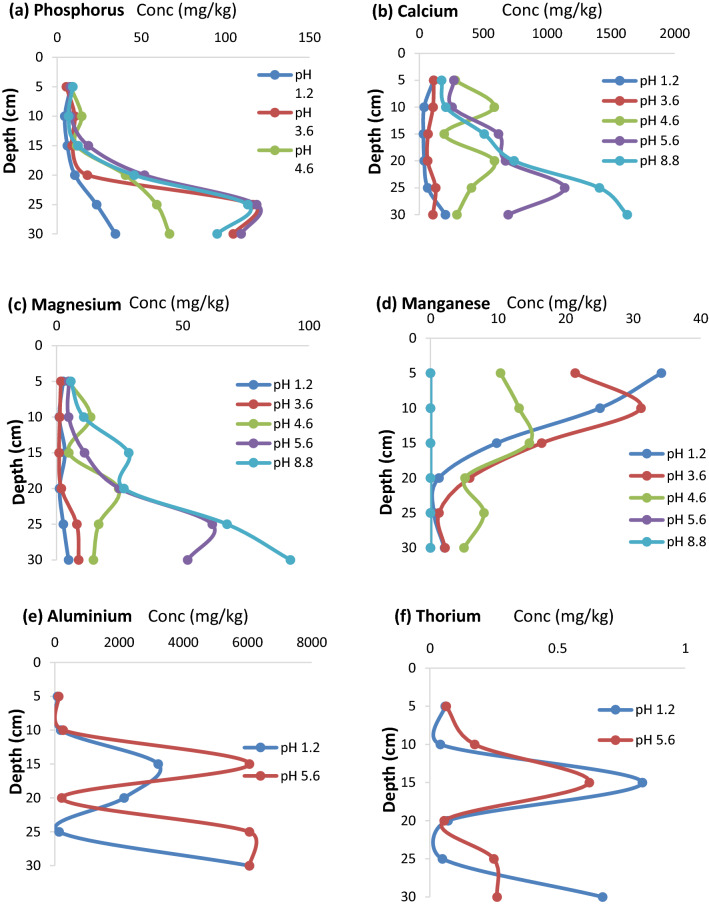
The concentration of residual ions in PG organic amended sandy soil with the depth of soil column after 30-day leaching with different of pH of lecahants.

After 30 days of leaching, P concentrations in PG organic soil column were higher at pH 3.6 to 8.8 leachants at the deeper soil column depth (Fig. 6a). In contrast, Ca concentration was found higher in the pH 8.8 leachant at the deeper part of the soil column, while its movement in the leachant of pH 1.2 and pH 3.6 was the least concentration observed (Fig. 6b). The same trend was observed as the case of Mg in Fig. 6c.

Different pattern of movement in the soil was observed in the case of metal ions classified as micro-nutrients, except for Fe that higher concentrations was found at the deeper part of the column, and this was peculiar to all the five leachants. Manganese was found in higher concentrations at the top of the column but lower at the bottom, except for the alkaline leachant, which all have been leached out into the leachate, no Mn was found left in the soil (Fig. 6d).

## Field evaluation of ions released from PG organic in a sandy soil

### PG organic application effect on soil

The soil is a sandy type, with particles dominated by more than 80% sand (Supplementary data available—IV). The organic matter (OM) content remained unchanged throughout the study period. Soil pH was strongly acidic but improved significantly at the last harvest from 4.11 to 5.64. The electrical conductivity (EC) and cation exchange capacity (CEC) remain unchanged throughout the study period, with value of ~ 2.0 mS/cm and < 2.0 cmol+ /kg soil, respectively. The CEC value was extremely low, which is typical for the sandy texture soil. The available P showed no significant difference before and after the PG organic treatment. Total C significantly increased from 1.7 to 3.47% after PG organic application; however, total N and S showed the opposite trend.

The content of cytotoxic metal ions As, Cd, Cr, Pb, Hg and Se in soil after PG organic application did not show a significant increase from the metal ion contents before PG organic applications throughout the study period (Supplementary data available—IV). All values were well below the target values stipulated in the New Dutch List^35^. Phytotoxic metals showed a significant (*p* < 0.05) increase in Zn and Mn contents, but not B. Zinc and Mn are plant micronutrients normally added to fertilizer as trace elements to improve plant growth and yield. The Zn content in the soil was lower than the target values provided by the New Dutch List^35^. For the content of other metals, such as Ce, Sr and La showed significantly higher contents in the maximum PG organic treatment at the second harvest compared to the baseline data but decreased significantly at the third harvest.

### PG organic application effect on water

The pH values of surface water and water in the boreholes were acidic (Supplementary data available—V). The pH of water in the borehole was within Class IV of the National Water Quality Standard^37^. The pH of surface water was below the Class IV range in the NWQS standard. Other in situ parameters fluctuated during different sampling times. This in situ parameter could be influenced by the amount of water in the borehole and stream due to changing seasons and the rising and falling of the tide. The salinity and EC readings in the boreholes were lower than the limit in the Class IV standard. Surface water recorded a high EC reading at the baseline compared to the Class IV standard. A high DO reading of more than 3 mg/L indicated that both boreholes and surface water were much better than Class IV in the NWQS standard.

The cation contents (Al, Ca, Mg and K) in the surface water and in the boreholes varied significantly with time of sampling. Metal ion contents in water were separated between the boreholes and surface water (Supplementary data available—V). This was to give a better data comparison to their respective values before PG organic application.

Concentrations of cytotoxic metal ions, As, Cd, Cr, Pb, Hg, and Se in water boreholes were generally significantly lower than those in the surface water (Supplementary data available—V). Their concentrations fluctuated with the time of sampling. All values were lower than the target value stipulated in the Malaysian Standard for Raw Drinking Water Quality^37^. Phytotoxic metal ions, Zn, Mn, and B concentrations fluctuated with the time of sampling. The contents of these metal ions in the boreholes were significantly lower than in the surface water. Their contents in the borehole water were not significantly different with the baseline data and lower than the target values in the Malaysian Standard for Raw Drinking Water Quality^40^. The other metal ions concentrations in boreholes also fluctuated with the time of sampling. Their concentrations in the boreholes were significantly lower than in the surface water.

### PG organic application effect on plant nutrition

Ions related to plant nutrients, such as macronutrients in control plants (Supplementary data available—VI) showed a significantly higher content (*p* < 0.05) or were not significantly different (*p* > 0.05) from the PG organic treated plant. Total N, C, and S contents also showed a similar level in treated and control plants. The macronutrient contents in corn grains treated with PG organic were not significantly different (*p* > 0.05) or significantly lower than (*p* < 0.05) the control (Supplementary data available -VI). Carbon, N and S follow a similar pattern. There were significant increases in K and P in the third harvest season compared to the second harvest season.

For cytotoxic metal ions, As, Cd, Cr, Pb, Hg and Se contents in plants treated with PG organic were not significantly different or significantly lower than (*p* < 0.05) the control (Supplementary data available—VI). Phytotoxic metal ions B, Zn, and Mn in plants treated with PG organic were significantly lower or not significantly different compared to the control plants. There was no significant difference in the contents of other metal ions between the plants treated with PG organic and the control plants.

Cytotoxic metal ions As, Cd, Pb, Cr, Hg and Se in the corn grain recorded a low value compared to the limit stipulated in the Malaysian Food Act^38^ and Food Regulation^39^ standards (Supplementary data available—VI). Phytotoxic metal ions B, Mn and Zn in the plots treated with PG organic were significantly higher than the control. Concentrations of the other metal ions were low, and in general, the contents of metal ions in grain from plots treated with PG organic were significantly lower (*p* < 0.05) than the control.

### PG organic application effect on natural radioactivity of soil

The analysis revealed that the radioactivity concentrations of ^226^Ra, ^228^Ra, and ^40^K in all concentrations in Malaysia’s samples (Supplementary data available—VII) were found to be much lower than the average soil (^226^Ra = 67.0 Bq/kg; ^226^Ra = 82.0 Bq/kg; ^238^U = 66 Bq/kg; and ^232Th^ = 82.0 Bq/kg), as reported by the United Nations Scientific Committee on the Effect of Atomic Radiation ^54^. There were variations in the concentrations of ^226^Ra, ^228^Ra and ^40^K in soil before and after the application of PG organic for each season, however, there were no significant differences (*p* > 0.05) in the natural radionuclide concentrations due to the PG organic treatment.

### PG organic application effect on natural radioactivity of water

The activity concentrations of ^226^Ra, ^228^Ra and ^40^K in water were fluctuated for each location (Supplementary data available—VIII). In general, the activity concentration of ^228^Ra for both seasons ranged from 0.3 to 3.9 Bq/L and 0.6 to 3.9 Bq/L, respectively. However, concentrations of natural radionuclides in samples for each sampling point (BH1 to BH5), showed there were no significant differences (*p* > 0.05) in ^226^Ra, ^228^Ra and ^40^K concentrations in water for both seasons.

### PG organic application effect on natural radioactivity of plant

There was a significant difference in the activity concentration for ^40^K in Guinea grass (2nd season) and kenaf (3rd season) between the control sample and treated sample (PG organic sample) (Supplementary data available—IX). The average concentrations of ^40^K in the control samples for Guinea grass leaf (2nd season) and kenaf stem (3rd season) were recorded to be 19.9 ± 8.5 and 44.8 ± 3.1 Bq/kg, respectively. On the other hand, the average activity concentrations of ^40^K in the samples treated with PG organic (Guinea grass leaf and kenaf stem) increased to 225.3 ± 1.2 and 111.7 ± 1.2 Bq/kg, respectively. In addition, most plant samples contain higher ^40^K concentrations compared to the concentrations of ^40^K in soil.

## Discussion

The dissolution of PG organic releases ions into soil solution. At equilibrium, the concentration of ions released into solution are in congruent to the chemical composition of the PG organic. The dissolution of PG organic depends on the characteristics of PG organic, the soil properties, and characteristics of solvent. The ions released may be adsorbed to the soil particles or organic materials, such as humic acid components and/or absorb by plant through the root systems and transported to the organs, such leaf and fruit. Any ions are not adsorbed and absorbed are subjected to leaching losses through the soil profile to the underground water, river and finally to the sea. Therefore, the mode(s) of ions released and their movements in the soil, water and plant ecosystems are very important aspect in assessment of PG organic for agricultural usage.

In general, PG organic comprises mainly gypsum (Calcium sulfate hydrate) (PDF 00-033-0311) and deploy the monoclinic geometry (SG: C2/C, 15) as confirmed by Powder X-ray diffraction (PXRD) diffractogram (Supplementary data available—I). The presence of sulfur, carbon, magnesium, and thorium are indicated in this XRD diffractogram. Thorium is in the form of ThO_2_, which has a cubic geometry (SG: *Fm3m*) with the lattice constants a = 5.597 Å; and concur to the reported value in literature for bulk ThO_2_ (PDF00-042-1462). Chemically, PG organic is a natural compound with pH about 7 and electrical conductivity (5.0 mS/cm) of about ten-fold lower than the sea water. The macro-nutrients such as P, Ca, and micro-nutrients Zn, Cu, B, Co, Mn and Mo are important for plant growth and development; and other metal ions contributed to soil and water contamination and plant and human toxicity are detected at very low levels (Table 2).

**Table 2:** Chemical characteristics of PG organic.

Parameter	Value
Moisture (%)	13.79 ± 0.052
pH_water_ (1:2.5)	7.16 ± 0.012
Conductivity (mS/cm)	4.96 ± 0.058
Cation exchange capacity (CEC) (cmol_+_/kg)	24.31 ± 2.325
Exchangeable Ca	65.60 ± 5.205
Exchangeable K	14.82 ± 1.209
Exchangeable Mg	51.04 ± 5.074
Exchangeable Na	1.85 ± 0.010
Nitrogen (%)	1.29 ± 0.027
Phosphorus (%)	2.62 ± 0.143
Potassium (%)	2.24 ± 0.030
Calcium (%)	9.56 ± 0.598
Magnesium (%)	2.99 ± 0.022
Water soluble P (mg/kg)	154.67 ± 12.837
Aluminium (%)	0.74 ± 0.056
Boron (%)	0.02 ± 0.003
Copper (%)	0/01 ± 0.001
Ferum (%)	5.93 ± 0.345
Manganese (%)	0.17 ± 0.007
Zinc (%)	0.04 ± 0.003
Arsenic (mg/kg)	1.51 ± 0.083
Cadmium (mg/kg)	0.33 ± 0.01
Cobalt (mg/kg)	1.64 ± 0.024
Chromium (mg/kg)	21.84 ± 3.90
Mercury (mg/kg)	nd
Molybdenum (mg/kg)	1.26 ± 0.089
Nickel (mg/kg)	106.85 ± 1.14
Lead (mg/kg)	53.58 ± 3.03
Selenium (mg/kg)	3.34 ± 0.45

MS ISO/IEC 1702; nd = not detected.

The ions released from PG organics vary with the acidity of leachants used and the extent of dissolution vary with time for each ion (Fig. 5). For instance, Sr, Cr, Th and Pb did not dissolve at any time at the pH of leachants < 5.6. Though existed variations between the ions and pH of leachants during this experiment, the quantity of ions released from PG organic showed that most ions were released at about 40% or more occurred at pH of leachants between 1.2 and 4.6 (Table 3). In contrast, Th was almost not dissolved at all at pH of most common soil at about 5.6. The solubility of ions in the present study are related to the water solubility and chemical reagents used (Supplementary data available—X) to estimate suitability of PR fertilizer for used in direct application for several countries^22^.

**Table 3:** Amount of PG organic applied, and percentage dissolved at the end of leaching.

Elements	Quantity Applied	Quantity Dissolved	Dissolution of elements^#^	pH of leachant
	mg	mg	%	
P	73.50	48.64	66.18	pH 1.2
Ca	848.05	623.53	73.52	pH 3.6
Mg	224.25	100.72	44.91	pH 4.6
Cu	1.02	0.78	76.47	pH 1.2
Fe	224.00	121.62	54.29	pH 1.2
Mn	13.65	9.95	72.88	pH 3.6
Zn	1.75	0.87	49.93	pH 1.2
Al	757.87	334.43	44.13	pH 1.2
Sr	946.05	2.401	0.25	pH 1.2
Cr	0.38	0.144	37.96	pH 1.2
Th	0.87	0.005	0.54	pH 5.6
Pb	1.46	0.726	49.77	pH 1.2

^#^D = QD/QA × 100%, where: D = % of element dissolved, QD = quantity dissolved, QA = quantity applied; QD = cumulative quantity dissolved at day 30 in amended column minus the cumulative quantity dissolved at day 30 in control expressed in mg; QA = calculated total amount of elements in the PG organic applied to each column expressed in mg.

The pattern of ions mobility in the soil column during this study supported the view that most of easily dissolved ions were translocated more at a deeper depth of soil column (e.g., P, Ca and Mg) and in contrast the less dissolved accumulated at the lower or in in the middle (e.g., Al and Th) of soil column depth (Fig. 6).

Under field evaluation, PG organic treated plots were subjected to plant growing condition and weather condition, especially rainfall. Any ions released excess than the plant requirement is subjected to migration to the underground water table. Most of the soil chemical properties remain consistent throughout this study period as compared to standard value used for comparison of soil (Supplementary data available—IV). However, a significant increase in C from 1.7 to 3.4% was observed in the PG organic treated plot attributed to the addition of organic matter in PG organic. However, total N and S showed the opposite trend, since these two elements are normally required by plant for growth and development.

The application of PG organic to the experimental plot could influence the metal ion concentrations in the boreholes but it had not influenced the metal ion contents in the river though the experimental site proximity to the river (Supplementary data available—V). Any changes in metal ion contents in the river were a direct contribution of the natural input and anthropogenic input from the surrounding environment. Evidence from the data gathered in this monitoring program showed that the application of PG organic did not accumulate and contaminate the borehole water.

The concentration of ions for plant nutrition and other metal ions showed no significant difference between control and in plot treated with PG organic during the period of this study (Supplementary data available—VI). However, ability of plant absorbs those ions are critical in the assessment of plant uptake of metal ions in edible parts, such as grain. Since, dissolved ions are absorbed by the root systems and transported to the other organs for growth and development, and for yield. The corn grain in the experimental plot treated with PG organic showed BAC values of less than 1, except for B and Mn (Table 3). This suggests that all metal ions do not have the potential to be accumulated in the plant. In contrast B and Mn have a BAC of more than 1, indicating very active uptake. These metal ions are micronutrients normally required by plants for their growth and development.

Contamination of soil is indicated by the I-geo index. The I-geo index showed that all metal ions in this site are in grade 0 (Table 4). This suggests that a sandy soil used at these experimental plots treated with PG organic were not contaminated. The CF values of all the metal ions in the study area were low (< 1) indicating that there was no increase of metal ions in the soil. In addition, the PLI values for all metal ions were very low, indicating that the soils were not polluted by the application of PG organic.

**Table 4:** Biological accumulation coefficients of metal ions in grains and soil contamination levels following treatment with PG organic in the experimental plots.

Metal	Treatment plot	Notes
As		
**BAC**	0.19	Moderate
I-geo	−2.89	Uncontaminated
CF	0.20	low CF
Cd		
**BAC**	0.75	Moderate
I-geo	−1.38	Uncontaminated
CF	0.58	low CF
Pb		
**BAC**	0.05	Low
I-geo	−0.36	Uncontaminated
CF	0.45	low CF
**BAC**	0.03	Low
I-geo	−3.86	Uncontaminated
CF	0.10	low CF
Se		
**BAC**	0.00	Weak
I-geo	0.00	Uncontaminated
CF	0.00	low CF
Zn		
**BAC**	0.61	Moderate
I-geo	−3.08	Uncontaminated
CF	0.18	low CF
Mn		
**BAC**	1.76	Active
Igeo	−4.29	Uncontaminated
CF	0.07	low CF
B		
**BAC**	20.88	Active
Igeo	−6.87	Uncontaminated
CF	0.01	low CF
PLI	0.25	Not polluted

Addition of PG organic to soils in the study area to enhance the growth and development and yield was proved to have no effect on the metal’s ion accumulation in soils. The experimental site pollution indices showed low values of I-geo, CF, and PLi. Subsequently, low BAC value in plant supports the hypothesis that the application of PG organic to the sandy soil deos not increase metal ions in plant or grain.

Since PG organic is derived from a mixture of WLP: NUF from rare-earth processing plant and organic materials, there is a possibility of radionuclides elements present in the PG by-products. The natural radioactivity of soil as measured by the concentration of ^226^Ra, ^228^Ra, and (Supplementary data available—VII) showed there was no significant difference between the plot treated with PG organic and control for the different season. The comparison between the previous studies performed in Malaysia showed that the results obtained in this study were in the normal range (Supplementary data available—VII). In Malaysia, the paddy soils values were 83.6 ± 40.4 for ^226^Ra, 108.1 ± 28.4 for ^228^Ra and 403.8 ± 224.8 for ^40^K Bq/kg. However, the respective values were slightly higher than reported in Egypt’s soil^55^. The differences in natural radionuclide concentrations in soil are related to several factors, such as geographical factors and geological conditions, as well as to the extent that fertilizer is used on the land in the agricultural industry ^56^. Prolong usage of fertilizer in the paddy soil in Malaysia attributed to the higher of these values as compared to the other soils. In addition, this study showed ^40^K had the highest concentrations compared to ^226^Ra and ^228^Ra. The higher ^40^K was ascribed to K as water soluble nutrient that tends to dissolve and accumulate on the soil surface ^56^. Since ^40^K is not from the uranium or thorium decay series^57^, its concentrations in soil do not contribute significantly to the total radioactive concentration in soil. Potassium-40 exists in nature, but the activity concentration may increase due to the use of fertilizer in agriculture.

The activity concentrations of ^226^Ra, ^228^Ra and ^40^K in water recorded from this study (Supplementary data available—VIII) were lower than the levels reported by Almayahi et al.^45^ in water samples sampled along the Northern Peninsula of Malaysia. The present study suggests that there was no leaching of the radionuclides from soil to groundwater. The activity concentrations of ^226^Ra, ^228^Ra and ^40^K in groundwater depended greatly on the type of minerals derived from aquifer rocks and soil compounds^58^. In addition, the surface water analysis from the nearest river also suggested that there is no accumulation (discharge) of natural radionuclides in the water due to the application of PG organic. This result is also in agreement to the concentration of metal ions measured in the water bore hole and the surface water samples (Supplementary data available—V).

Natural activity in plant and grain showed the results (Supplementary data available—IX) agree to previous studies^59–63^. This is not the expected result, since the activity concentrations of ^40^K in the soil samples treated with PG organic were much lower than the concentrations of ^40^K in the plant samples. The increases in the ^40^K concentration in plants (compared to the soil) might be contributed by other or additional sources, such as fertilizers, pesticides, or herbicides rather than PG organic. It should be noted that the uptake mechanism will differ for each plant species. Several factors, such as the kinetic parameters of plants to absorb specific radionuclides, metabolic behavior as well as plant tolerance for a specific radionuclide will influence the uptake of radionuclides by plants^64^. In addition, International Atomic Energy Agency, IAEA^65^ reported that the level of radioactivity concentrations for ^226^Ra and ^238^U in NPK fertilizer can reach up to 450 and 1280 Bq/kg, respectively. Therefore, further investigation is recommended to verify the sources that contribute to the increase of ^40^K in plants.

Though some variations were observed in the natural activity in the soil, however, the calculated radiological hazard index as measured by Ra_eq_ for soil was much lower than the 370 Bq/kg, a recommended limit of Ra_eq_ in soil (Table 5). For non-hazardous materials, the calculated Ra_eq_ should not exceed a maximum value of 370 Bq/kg for soil^66^. Therefore, the radiological concern due to the ^226^Ra, ^228^Ra and ^40^K in soil can be ignored; hence, PG organic is suitable for agriculture applications. The TFs of ^226^Ra, ^228^Ra, ^238U^, ^232^Th and ^40^K obtained in this study were comparable to those observed in several types of plants from other studies (Supplementary data available -IX). The highest radionuclide TF was ^40^K, which ranged from 0.85 to 6.98. Minor dissimilarities of the TF among the samples were perhaps due to transport processes as well as different absorption capacities of the plants itself^67^. It may also depend on the type of soil, soil texture, clay content, pH and organic matter content^68^.

**Table 5:** Radium equivalent and soil-to-plant transfer factor obtained in this study.

Season	Sample	Ra_eq_	Transfer factor soil-to-plants			Note
			^226^Ra	^228^Ra	^40^K	
			Bq/kg			
	Kenaf-Soil-Control	43.2	0.54	0.08	2.88	This research
	Kenaf-Soil-PG organic	46.6	0.71	0.03	5.34	
2nd season	Guinea grass-Soil-Control	44.7	0.10	0.01	0.30	
	Guinea grass -Soil-PG organic	67.0	0.12	0.01	3.75	This research
	Kenaf-Soil-Control	42.7	0.70	0.05	0.85	
	Kenaf-Soil-PG organic	50.7	0.42	0.01	2.12	
3rd season	Guinea grass -Soil-Control	41.3	0.50	0.12	6.98	
	Guinea grass -Soil-PG organic	45.0	0.28	0.02	3.54	
Rice (Malaysia)		–	0.04–0.20	0.02–0.15	0.09–4.14	^72^
Rice (Malaysia)		9.9–27.7	0.01–0.40	0.01–0.60	0.10–1.30	^73^
Vegetables		–	0.08–0.93	0.15–0.56	1.27–3.7	^74^
Vegetables (Malaysia)		–	0.03–0.09	0.001–0.01	1.59–5.20	^67^
Soya bean (Egypt)		–	0.21–0.57	–	0.95–2.52	^55^
Sesame and cowpea		–	0.25—0.56	–	1.04–1.54	^69^
Peninsular of Malaysia		63.2–414.4	–	–	–	^45^

## Conclusion

Both the phosphate fertilizer industry and a rare-earth processing facility are witnessing tremendous growth worldwide PG by-products, commonly stacked near their processing plants. For these by-products to be used in agriculture’s field, it must be proven not to have an adverse effect to the soil, water, and plant ecosystems. The ions released from PG organic under laboratory conditions showed that depends on the acidity of the lechant used. The pattern of ion ions released differed greatly under the same pH condition. Most ions are released under a very acid pH < 4.6 but less at the pH for optimum growth (pH 5.5 to 6.5). This is an ideal prevailing condition under field situation. Under field condition, the metal ion contents in borehole were significantly lower than the their contents in surface water. Metal ion contents in the borehole after PG organic application were below the target value standard in the Malaysian Standard for Raw Drinking Water Quality. Metal ion contents in soil in the plot treated with PG organic were significantly lower or not significantly different compared to the control. All metal ions concentrations were below the target values stipulated in the new Dutch list. Metal ion concentrations in the corn grain were lower than the standards in the Malaysian Food Act and Food Regulation.

The analysis of the naturally occurring radionuclide concentrations in soil samples indicated that the radioactivity concentrations of ^226^Ra, ^228^Ra, and ^40^K in all samples were much lower than their average concentrations in most soils as reported by United Nations. The determined radioactivity concentrations of ^226^Ra, ^228^Ra and ^40^K were also comparable to those published values in the literature. The analysis of the natural radioactivity concentration in several plants also showed that the results obtained in this study were comparable with those other studies. The analysis of the groundwater indicated that no leaching of the radionuclides from soil to groundwater occurred. In addition, the analysis of the surface water (nearest river) suggested that no accumulation (discharge) of natural radionuclide due to the application of PG organic occurred in the surface water. The calculated Ra_eq_ for soil was less than the 370 Bq/kg recommended limit of R_e_q in soil.

Based on these findings, it can be concluded that the application of PG organic had no impact on the environment (in soil and plants) at the studied location. The BAC, CF, I-geo and PLI showed that there was no accumulation of metal ions in grain, and no contamination and no pollution occurred in borehole water and the BRIS soil that received applications of PG organic. These data indicated that the use of PG organic as a soil conditioner in sandy soil is safe for the soil–plant-water ecosystem.

## Supplementary Information

Supplementary Information.
