# Supplementary material for: Valorization of rare earth processing byproducts for agriculture usage
Source: Sci Rep. 2021 Jul 27;11:15234. doi: 10.1038/s41598-021-93704-9 (PMC8316506; doi:10.1038/s41598-021-93704-9)
Supplement: Supplementary file 1 — Supplementary Information. [file 41598_2021_93704_MOESM1_ESM.docx]

**Valorization of rare earth processing byproducts for agricultural usage**

**Supplementary data – I**

**The XRD diffractogram of PG organic conditioner using PXRD method.**


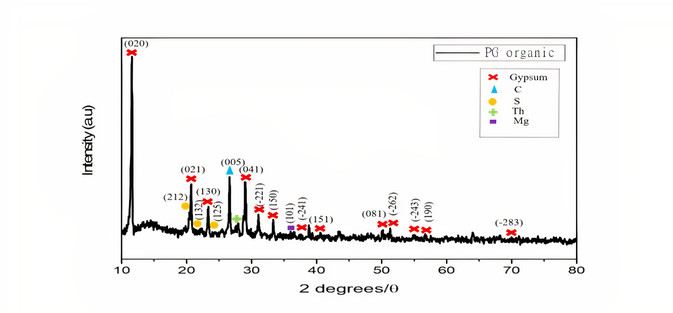


**Supplementary data – II**

**Comparison between the measured radioactivity concentrations and the recommended values by IAEA**

| Radionuclides | IAEA CRM-375 | | |
| --- | --- | --- | --- |
|  | Recommended value | Measured value | LLD |
|  | −−−−−−−−−−−−−−− Bq/kg −−−−−−−−−−−−−−−−−−−− | | |
| ^226^Ra (^238^U series) | 24.4^*^ (19 – 29.8)^**^ | 20.5 | 0.3 |
| ^228^ Ra (^232^Th series) | 20.5 (19.2 – 21.9) | 21.7 | 0.2 |
| ^40^K | 424 (417 – 432) | 430.6 | 1.2 |

*Note: ^*^ Certified value; ^**^ Certified value within 95% confidence level.*

| **Supplementary data – III**  **Cumulative quantity of ions dissolved at the different pH of leachants at the end of experiment.** | | | | | | | | |  |  |
| --- | --- | --- | --- | --- | --- | --- | --- | --- | --- | --- |
|  |  |  |  |  |  |  |  |  |  |  |
| pH of leachant | P | Ca | Mg | Cu | Fe | Mn | Zn |  |  |  |
| mg | | | | | | | |  |  |  |
| pH 1.2 | 57.63±1.00 a | 791.30±1.13 c | 100.92±1.00 b | 1.59±0.07 a | 165.22±1.07 a | 11.80±0.10 b | 4.15±0.01 a |  |  |  |
| pH 3.6 | 12.50±1.00 b | 883.99±1.15 a | 87.82±1.00 c | 0.09±0.01 b | 22.17±1.04 b | 11.99±0.06 a | 2.96±0.01 b |  |  |  |
| pH 5.6 | 0.44±1.10 d | 311.23±1.08 e | 106.76±6.73 a | 0.12±0.01 b | 4.04±0.14 d | 1.20±0.01 e | 0.09±0.01 d |  |  |  |
|  | Al | Sr | Cr | Th |  |  |  |  |  |  |
|  |  |  | mg |  |  |  |  |  |  |  |
| pH 1.2 | 658.69 | 29.06 | 0.40 | 0.01 | 1.53 |  |  |  |  |  |
| pH 5.6 | 3.94 | 21.50 | 0.35 | 0.01 | 0.01 |  |  |  |  |  |
| p-value | 0.001 (^**^) | 0.004 (^**^) | 0.001 (^**^) | 0.008 ^(**^) | 0.02 (^*^) |  |  |  |  |  |
| *Note: Values are means as compared with paired t-test statistic; asterisk symbol ^(*^) means significant at p≤0.05 and (^**^) means highly significant at p≤0.01.* | | | | | | | | | | |

**Supplementary data – IV**

**Soil properties before and after PG organic application in the experimental plot.**

| Sample | 22-05-2015 | 22-01-2016 | 27-09-2016  Harvest 1 | | 08-12-2016  Harvest 2 | | 24-04-2017  Harvest 3 | |  |
| --- | --- | --- | --- | --- | --- | --- | --- | --- | --- |
|  | PG organic application | | Control | Max | Control | Max | Control | Max |  |
|  | Before  (baseline) | After |  |  |  |  |  |  |  |
| Sand (%) | 87 | 90 | na | na | 90 | 91 | 84 | 85 |  |
| Silt (%) | 8 | 4 | na | na | 7 | 5 | 9 | 8 |  |
| Clay (%) | 5 | 6 | na | na | 3 | 4 | 7 | 7 |  |
| Texture | Sand | Sand | na | na | Sand | Sand | Sand | Sand |  |
| Organic matter (%) | 6.14 | 5.45 | 5.02 | 5.25 | 6.10 | 5.78 | 5.60 | 5.64 |  |
| pH water | 4.11 | 4.63 | 5.13 | 5.34 | 4.15 | 4.25 | 5.79 | 6.08 |  |
| EC (mS/cm) | 2.04 | 2.05 | 1.87 | 1.96 | 1.94 | 1.94 | 1.99 | 2.10 |  |
| Exch Al (cmol^+^/kg) | 0.47 | 0.04 | na | na | 0.01 | 0.01 | 0.05 | 0.04 |  |
| Exch H (cmol^+^/kg) | 0.33 | 0.01 | na | na | 0.02 | 0.01 | 0.04 | 0.03 |  |
| Exch Ca (cmol^+^/kg) | 0.34 | 0.59 | na | na | 1.53 | 1.70 | 0.16 | 0.22 |  |
| Exch Mg (cmol^+^/kg) | 0.34 | 0.37 | na | na | 0.86 | 1.01 | 0.71 | 0.69 |  |
| Exch Na (cmol^+^/kg) | 0.19 | 0.11 | na | na | 1.52 | 1.18 | 0.22 | 0.36 |  |
| Exch K (cmol^+^/kg) | 0.09 | 0.30 | na | na | 0.45 | 0.46 | 0.09 | 0.09 |  |
| CEC (cmol^+^/kg) | 1.75 | 1.41 | na | na | 4.38 | 4.36 | 1.20 | 1.42 |  |
| Available P (mg/kg) | 2.44 | 5.35 | na | na | 1.60 | 1.39 | 1.90 | 2.04 |  |
| Total C (%) | 1.70 | 3.17 | 2.92 | 3.05 | 16.80 | 15.17 | 2.30 | 2.48 |  |
| Total N (%) | 1.16 | 1.05 | 0.16 | 0.02 | 0.28 | 0.28 | 0.10 | 0.22 |  |
| Total S (%) | 0.13 | 0.13 | 0.01 | 0.02 | 0.12 | 0.09 | 0.02 | 0.02 |  |
| C/N ratio | 1.47 | 3.02 | 18.25 | 152.50 | 59.36 | 54.40 | 22.35 | 13.02 |  |
| Metal ions  (mg/kg) |  |  |  |  |  |  |  |  | Target Value (mg/kg) |
| Total As | 4.03b | 2.97bc | 2.24cd | 1.49d | ND | ND | 3.15b | 5.54a | 29 |
| Total Cd | 0.09c | 0.07c | 0.14b | 0.06c | 0.29a | 0.25a | 0.08c | 0.08c | 0.8 |
| Total Cr | - | 11.04a | 8.53b | 5.52c | BDL | BDL | 6.61c | 7.96b | 100 |
| Total Pb | - | 10.41c | 27.48a | 16.65b | 20.72b | 19.76b | 11.22c | 11.71c | 50 |
| Total Hg | - | 0.06a | ND | ND | ND | ND | 0.03b | 0.02b | 0.5 |
| Total Se | 0.27c | 0.85a | ND | ND | 0.44c | 0.34c | 0.41c | 0.41c | 0.7 |
| Extractable B | 0.14d | 0.09d | 1.33c | 1.26c | 7.30a | 3.35b | 0.11d | 0.12d | - |
| Total Zn | 21.16d | 18.53d | 10.48e | 9.30e | 35.09b | 26.80c | 34.47b | 40.31a | 140 |
| Total Mn | 21.43e | 41.98c | 32.39d | 33.89d | 54.69b | 87.74a | 22.55e | 44.47c | - |
| Total Ce | 4.03f | - | 12.73e | 116.19b | 20.84d | 197.89a | 11.06e | 87.92c | - |
| Total La | 1.96e | - | 6.46d | 70.80b | 12.60c | 137.43a | 3.82d | 53.95b | - |
| Total Sr | 8.38c | 12.57c | 15.73c | 20.73b | 14.45c | 29.91a | 8.19d | 11.74c | - |
| Total Ag | - | 0.02b | 0.06a | 0.03b | BDL | BDL | 0.07a | 0.03b | - |
| Total Ba | - | 33.00a | 13.33c | 13.14c | 8.82d | 21.71b | 4.30e | 28.55a | 200 |

*Note: ND, not detectable; Source of Standard: The new Dutch list ^1^: In a row, means followed by a common letter are not significantly different at the 5% level.*

**Supplementary data – V**

**Chemical properties and ions contents in boreholes and surface water before and after PG organic application.**

| Parameters | Baseline | | PG organic application | | | | | | | | Class IV  NWQS |
| --- | --- | --- | --- | --- | --- | --- | --- | --- | --- | --- | --- |
|  | 22-05-2015 | | 22-01-2016 | | Harvest 1  17-09-2016 | | Harvest 2  08-12-2016 | | Harvest 3  10-05-2017 | |  |
|  | Borehole | Surface Water | Borehole | Surface Water | Borehole | Surface Water | Borehole | Surface Water | Borehole | Surface Water |  |
| pH | 4.70bc | 5.08b | 6.10a | 4.44c | 5.89a | 4.29c | 5.69a | 3.96d | 4.93b | 5.04b | 5-9 |
| Temp (^o^C) | - | - | 24.96bc | 24.35c | 25.70b | 25.23b | 25.23b | 24.58c | 29.47a | 30.12a | - |
| EC (mS/cm) | 0.06ef | 6.52b | 0.04f | 0.08e | 0.05f | 82.00a | 0.07e | 0.40c | 0.11d | 0.30c | 6 |
| TDS (g/L) | - | - | 26.00e | 51.50d | 84.40c | 237.50a | 0.05g | 0.26f | 65.60d | 176.50b | 4000 |
| Salinity (%) | - | - | 0.02d | 0.04cd | 0.06c | 0.20a | 0.03d | 0.20a | 0.05c | 0.13b | 2 |
| DO (mg/L) | - | - | 2.98d | 4.53c | 4.52c | 5.45b | 5.37b | 2.93d | 5.61b | 7.58a | < 3 |
| Al (µg/L) | 37.43g | 804.64a | 12.42h | 631.10b | 97.93f | 420.54d | 48.35g | 554.07c | 261.59e | 607.21b | NL |
| Ca (µg/L) | 2681.89b | 33978.22a | 2229.52b | 1827.40c | 1149.86d | 2626.90b | 1074.82d | 707.21e | 1285.22d | 781.52e | NL |
| Mg (µg/L) | 667.20g | 193666.85a | 684.64g | 1151.90f | 1904.50e | 3679.46d | 766.83g | 537.17g | 10595.62b | 6958.86c | NL |
| K (µg/L) | 1543.86e | 42289.70a | 1736.70e | 1656.00e | 2614.78d | 5648.17b | 1289.16e | 828.94f | 5989.34b | 3481.78c | NL |
| Na (µg/L) | - | - | - | - | 4041.91c | 6010.91b | 1335.10f | 2012.10e | 2700.59d | 94031.11a | NL |
| Metal ions (µg/L) | |  |  |  |  |  |  |  |  |  | *Target value (µg/L) |
| As | 0.30e | 4.08a | 0.05g | 0.85d | 0.21f | 0.99d | 0.86d | 2.81b | 0.18f | 1.40c | 10 |
| Cd | 0.01e | 0.17c | 0.02e | 1.04a | 0.04d | 0.42b | 0.04d | 0.03d | 0.05d | 0.02e | 0.3 |
| Cr | - | - | 0.61d | 3.59a | 0.38d | 4.06a | 0.19e | 1.84c | 2.02c | 3.02b | 50 |
| Pb | - | - | 0.39d | 3.56b | 0.70c | 4.52b | ND | ND | 7.27a | 0.68c | 10 |
| Hg | - | - | 0.06b | 0.05b | ND | ND | ND | ND | 1.15a | 1.09a | 1 |
| Se | 0.24d | 4.18a | 0.25d | 0.15e | 0.42c | 1.73b | 0.17e | 0.22d | 3.25a | 1.85b | 10 |
| B | 34.83c | 272.73a | 18.82d | 24.76d | 11.31e | 50.63b | 7.31f | 19.60d | 18.20d | 49.67b | - |
| Zn | 45.10d | 101.70b | 51.97d | 81.99c | 54.67d | 198.75a | 19.70f | 51.11d | 29.61e | 80.79c | 3000 |
| Mn | 19.44d | 82.80b | 5.08e | 32.25c | 26.70c | 398.57a | 8.94e | 25.92c | 23.65cd | 80.61b | 200 |
| Th | 0.03f | 0.26d | 0.68c | 36.75b | 0.68c | 105.85a | 0.01f | 0.33d | 0.17e | 0.87c | - |
| Ce) | 0.11g | 0.38f | 10.80c | 226.73a | 0.34f | 43.83b | 0.16g | 1.36e | 5.37d | 5.23d | - |
| La | 0.04h | 0.23g | 10.14d | 183.78a | 46.68c | 119.34b | 0.08h | 0.56f | 1.81e | 1.66e | - |
| Sr | 22.32c | 725.65a | 27.13c | 16.44d | 0.00g | 1.59f | 24.08c | 9.90e | 62.64b | 58.37b | - |
| Ag | - | - | 0.03b | 0.17a | 0.00c | 0.05b | 0.00c | 0.00c | ND | ND | - |
| Ba | - | - | 99.77b | 102.13b | 79.70c | 163.48a | 35.87e | 53.44d | 90.49b | 168.62a | 50 |

*Note: ND, not detectable; Source of standard: *Malaysian Standard for Raw Drinking Water Quality ^2^; The new Dutch list ^1^; In a row, means followed by a common letter are not significantly different at the 5% level.*

**Supplementary data – VI**

**Nutrient and metal ions contents of cultivated plants and corn grain in the experimental plots.**

| Parameter (Plants) | Sampling frequency | | | | | | |
| --- | --- | --- | --- | --- | --- | --- | --- |
|  | 27-09-2016 | | 08-12-2016 | | | 24-04-2017 | |
|  | Control | Max | Control | | Max | Control | Max |
| Total Ca (mg/kg) | 1934.67a | 914.70b | 1981.95a | | 1909.94a | NPG | 1651.62a |
| Total Mg (mg/kg) | 1306.91c | 1966.74b | 10862.66a | | 9402.54a | NPG | 1987.68b |
| Total K (mg/kg) | 5164.62a | 3433.87b | 6877.75a | | 2271.21c | NPG | 1745.17d |
| Total Al (mg/kg) | - | - | 92.86a | | 53.00b | NPG | 78.06a |
| Total P (mg/kg) | 945.30b | 829.45b | 1968.21a | | 2043.75a | NPG | 791.64c |
| Total C (%) | - | - | 40.30a | | 40.23a | NPG | 39.70a |
| Total N (%) | - | - | 1.26a | | 1.28a | NPG | 1.27a |
| Total S (%) | - | - | 0.39a | | 0.39a | NPG | 0.38a |
| C/N ratio | - | - | 31.89a | | 31.52a | NPG | 31.26a |
| Parameter (corn grain) | 27-09-2016 | | 08-12-2016 | | | 24-04-2017 | |
|  | Control | Max | Control | Max | | Control | Max |
| Total Ca (mg/kg) | - | - | 1879.24a | 1449.51b | | 27.50c | 33.01c |
| Total Mg (mg/kg) | - | - | 1964.66a | 1954.59a | | 735.95b | 820.60b |
| Total K (mg/kg) | - | - | 2333.09b | 1658.17c | | 15301.32a | 16092.46a |
| Total Al (mg/kg) | - | - | 75.67a | 79.06a | | 28.27b | 11.04c |
| Total P (mg/kg) | - | - | 1587.28b | 695.60c | | 2759.50a | 3240.87a |
| Total C (%) | - | - | 40.21a | 39.61a | | 33.25b | 34.98b |
| Total N (%) | - | - | 1.26a | 1.27a | | 0.75c | 0.93b |
| Total S (%) | - | - | 0.39a | 0.39a | | 0.08c | 0.12b |
| C/N ratio | - | - | 31.88 | 31.14 | | 44.53 | 37.48 |

| Metal ions (Plants) (mg/kg) | Sampling frequency | | | | | |  |
| --- | --- | --- | --- | --- | --- | --- | --- |
|  | 27-09-2016 | | 08-12-2016 | | 24-04-2017 | |  |
|  | Control | Max | Control | Max | Control | Max |  |
| Total As | 0.68a | 0.62a | ND | ND | NPG | 0.10 |  |
| Total Cd | 0.07b | 0.09b | 0.21a | 0.22a | NPG | 0.03c |  |
| Total Pb | 0.86b | 0.64c | 2.22a | 0.98b | NPG | 0.24d |  |
| Total Cr | 0.38b | 0.33b | ND | ND | NPG | 1.94a |  |
| Total Hg | 0.02a | 0.01a | ND | ND | NPG | ND |  |
| Total Se | 0.18a | 0.13b | 0.05c | 0.04c | NPG | 0.06c |  |
| Extractable B | 29.81a | 15.55b | 26.64a | 33.96a | NPG | 6.25c |  |
| Total Zn | 142.33a | 71.47b | 35.37c | 39.79c | NPG | 9.17d |  |
| Total Mn | 15.59d | 4.17e | 92.98a | 63.99b | NPG | 49.64c |  |
| Total Ce | 1.47b | 2.08b | 0.34c | 0.30c | NPG | 5.39a |  |
| Total La | 0.97a | 1.54a | 0.34b | 0.35b | NPG | 0.16c |  |
| Total Sr | 16.63b | 9.21c | 58.49a | 68.06a | NPG | 11.18c |  |
| Total Th | 0.13b | 0.12a | ND | ND | NPG | 0.08c |  |
| Total Ag | 0.02a | 0.00b | ND | ND | NPG | ND |  |
| Total Ba | 2.21c | 1.11d | 10.28a | 8.29b | NPG | 1.08d |  |
| Metal ions (Corn grain) (mg/kg) | 27-09-2016 | | 08-12-2016 | | 24-04-2017 | | ^*^Limit  mg/kg |
|  | Control | Max | Control | Max | Control | Max |  |
| Total As | - | - | 0.02c | ND | 0.37a | 0.22b | < 1 |
| Total Cd | - | - | 0.00 | 0.36a | 0.01a | 0.01a | < 1 |
| Total Pb | - | - | 0.07c | 0.30a | 0.44a | 0.30a | < 2 |
| Total Cr | - | - | 1.43c | ND | 3.17b | 4.55a | - |
| Total Hg | - | - | ND | ND | ND | ND | - |
| Total Se | - | - | 0.01b | 0.03a | 0.04a | 0.02ab | < 0.05 |
| Extractable B | - | - | 2.82b | 2.24b | 23.88a | 20.49a | - |
| Total Zn | - | - | 10.63c | 18.63b | 40.91a | 38.12a | - |
| Total Mn | - | - | 7.52b | 5.67c | 6.69bc | 9.91a | - |
| Total Ce | - | - | 0.29c | 0.20c | 1.38a | 0.69b | - |
| Total La | - | - | 0.07d | 0.23c | 0.70a | 0.47b | - |
| Total Sr | - | - | 1.47a | 0.80d | 1.07b | 0.94c | - |
| Total Th | - | - | 0.02 | ND | ND | ND | - |
| Total Ag | - | - | ND | ND | ND | ND | - |
| Total Ba | - | - | ND | ND | 1.06a | 0.97a | - |

*Note: ND: not detected; max: maximum treatment; NPG: no plant growing; source of standard: *Malaysian Food Act ^3^ and Food Regulation ^4^; within rows, means followed by a common letter are not significantly different using LSD test at the 5% level of significance.*

**Supplementary data – VII**

**Comparison of radioactivity concentrations of ^226^Ra, ^228^Ra and ^40^K in soil samples obtained in this study with other literature.**

| Season | Sample ID |  | Activity Concentration (Bq/kg) | | |
| --- | --- | --- | --- | --- | --- |
|  |  |  | ^226^Ra | ^228^Ra | ^40^K |
| 2^nd^ Season | Kenaf-Soil-Control | Range | 13.8 - 17.6 | 15.7 - 16.8 | 44.5 - 47.0 |
|  |  | Average | 15.7 ± 2.7 | 16.3 ± 0.8 | 45.7 ± 1.8 |
|  | Kenaf-Soil-Condi | Range | 14.0 - 16.0 | 18.0 – 19.9 | 46.7 - 49.7 |
|  |  | Average | 15.0 ± 1.4 | 19.0 ± 1.4 | 48.2 ± 2.1 |
|  | ANOVA Analysis | | 0.77^ns^ | 0.13 ^ns^ | 0.12 ^ns^ |
|  | Guinea grass-Soil-Control | Range | 14.4 - 16.8 | 15.6 - 17.1 | 59.6 - 72.8 |
|  |  | Average | 15.6 ± 1.7 | 16.3 ± 1.1 | 66.2 ± 9.3 |
|  | Guinea grass-Soil-PG organic | Range | 15.4 - 17.5 | 27.6 - 34.7 | 54.8 - 65.4 |
|  |  | Average | 16.5 ± 1.5 | 31.2 ± 5.0 | 60.1 ± 7.5 |
|  | ANOVA Analysis | | 0.70^ns^ | 0.06^ns^ | 1.00^ns^ |
| 3^rd^ Season | Kenaf-Soil-Control | Range | 13.3 - 14.7 | 16.5 - 17.0 | 49.3 - 55.5 |
|  |  | Average | 14.0 ± 1.0 | 16.8 ± 0.4 | 52.4 ± 4.4 |
|  | Kenaf-Soil-PG organic | Range | 16.7- 17.6 | 19.2 - 21.0 | 47.8 - 57.7 |
|  |  | Average | 17.1 ± 0.6 | 20.1 ± 1.3 | 52.8 ± 7.0 |
|  | ANOVA Analysis | | 0.07 ^ns^ | 0.07 ^ns^ | 0.96 ^ns^ |
|  | Guinea grass-Soil-Control | Range | 12.2 - 14.8 | 16.0 - 16.7 | 47.7 - 53.2 |
|  |  | Average | 13.5 ± 1.8 | 16.3 ± 0.4 | 50.4 ± 3.9 |
|  | Guinea grass-Soil-PG organic | Range | 13.7 - 15.1 | 16.8 - 18.1 | 60.6 - 61.4 |
|  |  | Average | 14.4 ± 1.0 | 17.6 ± 0.7 | 61.0 ± 0.6 |
|  | ANOVA Analysis | | 0.60 ^ns^ | 0.05^*^ | 0.06 ^ns^ |
| Malaysia’s Soil ^5^ | | | 67.0 | 82.0 | 310 |
| Palm Oil Plantation, Malaysia ^6^ | | | 21.5 ± 8.4 | 29.2 ± 8.9 | 56.8 ± 36.3 |
| Paddy Plantation, Malaysia ^7^ | | | 83.6 ± 40.4 | 108.1 ± 28.4 | 403.8 ± 224.8 |
| Agriculture soil, Egypt ^8^ | | | 21.2 ± 0.9 | - | 194± 9.5 |
| Kuala Krai, Kelantan ^9^ | | | 40.2 – 264.0 | 29.2 – 312.9 | 491.1 – 1184.2 |

*Note: ns: not significant; ^*^ and ^**^: significant at p ≤ 0.05 and p ≤ 0.01, respectively.*

**Supplementary data – VIII**

**Activity concentrations of ^226^Ra, ^228^Ra and ^40^K in water obtained from studied location.**

| Season | Sample ID |  | Activity Concentration (Bq/L) | | |
| --- | --- | --- | --- | --- | --- |
|  |  |  | ^226^Ra | ^228^Ra | ^40^K |
| 2^nd^ Season | BH-1 | Range | 0.5 - 0.9 | 1.1 – 3.9 | 6.9 - 8.0 |
|  |  | Average | 0.7 ± 0.3 | 2.5 ± 2.0 | 7.4 ± 0.8 |
|  | BH-2 | Range | 0.4 - 0.5 | 0.9 - 1.1 | 1.8 - 9.8 |
|  |  | Average | 0.5 ± 0.1 | 1.0 ± 0.1 | 5.8 ± 5.6 |
|  | BH-3 | Range | BDL - 0.3 | 0.4 - 0.6 | 1.4 - 1.8 |
|  |  | Average | 0.3 ± 0.0 | 0.5 ± 0.1 | 1.6 ± 0.3 |
|  | BH-4 | Range | 0.3 - 0.5 | 0.7 - 0.8 | 0.4 - 7.2 |
|  |  | Average | 0.4 ± 0.2 | 0.8 ± 0.1 | 3.4 ± 5.4 |
|  | BH-5 | Range | BDL | 0.3 - 0.6 | 3.3 - 5.1 |
|  |  | Average | DBL | 0.5 ± 0.2 | 4.2 ± 1.3 |
|  | **Average for BH** | | 0.4 | 1.1 | 4.5 |
|  | SW-1 | Range | BDL - 0.3 | 0.6 - 0.9 | 2.5 - 2.9 |
|  |  | Average | BDL | 0.8 ± 0.2 | 2.7 ± 0.3 |
|  | SW-2 | Range | 0.3 - 0.7 | 1.3 - 1.8 | 2.9 - 6.5 |
|  |  | Average | 0.5 ± 0.3 | 1.5 ± 0.4 | 4.7 ± 2.6 |
|  | **Average for SW** | | 0.4 | 1.2 | 3.7 |
| 3^rd^ Season | BH-1 | Range | BDL - 0.3 | 1.6 - 3.2 | 6.5 - 11.6 |
|  |  | Average | 0.2 ± 0.1 | 2.4 ± 1.1 | 9.0 ± 3.6 |
|  | BH-2 | Range | 0.3 - 0.4 | 0.6 - 2.3 | 7.6 - 8.0 |
|  |  | Average | 0.3 ± 0.0 | 1.5 ± 1.2 | 7.8 ± 0.3 |
|  | BH-3 | Range | 0.3 - 0.4 | 0.9 - 1.7 | 2.2 - 3.6 |
|  |  | Average | 0.4 ± 0.0 | 1.3 ± 0.5 | 2.9 ± 1.0 |
|  | BH-4 | Range | 0.4 - 0.6 | 1.8 - 2.0 | 5.4 - 5.8 |
|  |  | Average | 0.5 ± 0.2 | 1.9 ± 0.1 | 5.6 ± 0.3 |
|  | BH-5 | Range | BDL | 0.7 - 1.1 | 2.2 - 5.8 |
|  |  | Average | 0.1^*^ ± 0.0 | 0.9 ± 0.3 | 4.0 ± 2.6 |
|  | **Average for BH** | | 0.3 | 1.6 | 5.9 |
|  | SW-1 | Range | 0.3 - 0.4 | 1.7 - 2.9 | 6.1 - 10.1 |
|  |  | Average | 0.4 ± 0.0 | 2.3 ± 0.9 | 8.1 ± 2.8 |
|  | SW-2 | Range | BDL | 0.5 - 1.3 | 4.7 - 5.8 |
|  |  | Average | BDL | 0.9 ± 0.6 | 5.2 ± 0.8 |
|  | **Average for SW** | | 0.3 | 1.5 | 6.6 |
|  | **ANOVA analysis for BH for both seasons** | | 0.31^ns^ | 0.27 ^ns^ | 0.38 ^ns^ |
|  | **ANOVA analysis for SW for both seasons** | | 1.00 ^ns^ | 0.62 ^ns^ | 0.24 ^ns^ |
| 16 water sample at Norther Peninsular Malaysia ^10^ | | | 0.9 – 7.0 | 0.6 – 8.6 | 53.0 – 222.0 |
| Tap, stream, lake, river water at Seberang Perai, Malaysia ^11^ | | | 3.3 – 4.3 | 0.9 – 2.2 | 134.8 – 172.4 |
| 10 drinking water samples at Malaysia ^12^ | | | 0.41 – 0.56 | 0.34 – 0.99 | 2.88 – 7.65 |

*Note: ND: Not Detected****;*** *ns: not significant; Source of standard ^3^: ^*^The reported value is below the detection limit; Lower limit detection (LLD): ^226^Ra (0.3 Bq/L); ^228^Ra (0.1 Bq/L); ^40^K (1.2 Bq/L).*

**Supplementary data – IX**

## The radioactivity concentrations of ^226^Ra, ^228^Ra and ^40^K in plant samples.

| Season | Sample ID |  | Activity Concentration (Bq/kg) | | |
| --- | --- | --- | --- | --- | --- |
|  |  |  | ^226^Ra | ^228^Ra | ^40^K |
| 2^nd^ Season | Kenaf-stem-Control | Range | 8.3 – 8.5 | 1.1 - 1.5 | 116.8 - 146.4 |
|  |  | Average | 8.4 ± 0.1 | 1.3 ± 0.3 | 131.6 ± 20.9 |
|  | Kenaf-stem-PG organic | Range | 10.2 - 18.5 | 0.3 - 0.7 | 192.9 - 321.8 |
|  |  | Average | 10.6 ± 11.3 | 0.5 ± 0.3 | 257.3 ± 91.1 |
|  | ANOVA Analysis | | 0.19 ^ns^ | 0.10 ^ns^ | 0.20 ^ns^ |
|  | Guinea grass-leaf-Control | Range | 1.4 - 1.9 | 0.1 - 0.3 | 13.9 – 25.9 |
|  |  | Average | 1.6 ± 0.4 | 0.2 ± 0.2 | 19.9 ± 8.5 |
|  | Guinea grass-leaf-PG organic | Range | 1.8 - 1.9 | 0.1 - 0.6 | 224.4 - 226.2 |
|  |  | Average | 1.9 ± 0.0 | 0.4 ± 0.3 | 225.3 ± 1.2 |
|  | ANOVA Analysis | | 0.51 ^ns^ | 0.57 ^ns^ | 0.0009^**^ |
| 3^rd^ Season | Kenaf-stem-Control | Range | 7.2 - 12.4 | 0.6 - 1.2 | 42.7 - 47.0 |
|  |  | Average | 9.8 ± 3.7 | 0.9 ± 0.4 | 44.8 ± 3.1 |
|  | Kenaf-stem-PG organic | Range | 5.0 - 9.5 | 0.1 - 0.4 | 110.9 - 112.5 |
|  |  | Average | 7.2 ± 3.2 | 0.2 ± 0.2 | 111.7 ± 1.2 |
|  | ANOVA Analysis | | 0.54 ^ns^ | 0.16 ^ns^ | 0.001^**^ |
|  | Guinea grass-leaf-Control | Range | 4.8 – 8.5 | 1.2 - 2.7 | 321.0 - 384.4 |
|  |  | Average | 6.7 ± 2.6 | 1.9 ± 1.1 | 352 ± 44.9 |
|  | Guinea garss-leaf-PG organic | Range | 2.9 – 5.1 | 0.4 - 0.5 | 204.9 - 227.6 |
|  |  | Average | 4.0 ± 1.6 | 0.4 ± 0.1 | 216.2 ± 16.0 |
|  | ANOVA Analysis | | 0.34 ^ns^ | 0.18 ^ns^ | 0.06 ^ns^ |
| Vegetables (Malaysia) 13 | | | 1.3 – 7.8^#^ | 0.4 – 4.1^##^ | - |
| Vegetable leaves (Malaysia) ^14^ | | | 0.6 – 5.64 | - | 398.9 – 1072.6 |
| Corn (Malaysia) ^15^ | | | 0.1 – 19.2 | 0.1 – 3.2 | 26.1 – 129.0 |
| Paddy grain (Malaysia) ^16^ | | | 0.5 – 2.8 | 0.5 – 1.6 | 43.5 – 108.5 |
| Vegetables (Iraq) ^17^ | | | 4.0 – 7.0^#^ | 2.2 – 10.6^##^ | 109.0 – 319.2 |
| Soya bean (Egypt) 1^8^ | | | 2.3 – 9.0 | - | 140.0 – 270.0 |

*Note: ^#^Assume as ^226^Ra (^238^U decay series) in secular equilibrium; ^##^Assume as ^228^Ra (^232^Th decay series) in secular equilibrium. ns: not significant; ^*^ and ^**:^ significant at p ≤ 0.05 and p ≤ 0.01, respectively.*

**Supplementary data – X**

**The solubility of PG organic in reagents.**

| Elements | Solubility in | | | |
| --- | --- | --- | --- | --- |
|  | Water | 2% formic | 2% citric | Na acetate |
|  | −−−−−−−−−−−−− % −−−−−−−−−−−−−−−− | | | |
| P | 0.06 | 0.10 | 1.48 | 0.07 |
| Ca | 26.18 | 36.95 | 42.20 | 33.65 |
| Mg | 25.10 | 32.70 | 37.32 | 30.83 |
| Cu | 0.34 | 1.24 | 5.79 | 1.37 |
| Fe | 0.02 | 0.06 | 17.03 | 0.06 |
| Mn | 0.77 | 1.65 | 24.93 | 1.55 |
| Zn | 0.60 | 1.68 | 15.92 | 1.76 |
| Al | 0.02 | 0.01 | 0.99 | 0.01 |
| Sr | 0.17 | 0.12 | 0.13 | 0.12 |
| Th | 0.01 | 0.01 | 4.71 | 0.02 |
| Note: Element soluble in reagents over total amount detected in *aqua-regia* reagent. Data for K, Cr and Pb were not analyzed. | | | | |

**References**

1. Target D. Circular on target values and intervention values for soil remediation. The new Dutch list, version February 4^th^ (2000).
2. National Water Quality Standards (Annexe). Department of Environment, Ministry of Environment and Water. https://www.doe.gov.my/portalv1/wp-content/uploads/2019/05/Standard-Kualiti-Air-Kebangsaan.pdf. (2020).
3. MALAYSIA L.O. Food Act 1983. in: Act 281. 1–47 (1983).
4. MALAYSIA L.O. Food Regulations 1–123 (1985).
5. UNSCEAR. Exposures from Natural Radiation Sources. United Nations Scientiﬁc Committee on the Effects of Atomic Radiation. Report to General Assembly, With Annexes. United Nations, New York (2000).
6. Alias M., Hamzah Z., Saat A., Omar M. & Tajuddin Z. Determination of ^226^Ra, ^228^Ra and ^40^K in soil from felda Jengka-15 oil palm plantation*. Malays. J. Anal. Sci*. **9**, 126-132 (2005).
7. Alsaffar, M. S., Jaafar, M. S., Kabir, N. A. & Ahmad, N. Distribution of ^226^Ra, ^232^Th, and ^40^K in rice plant components and physico-chemical effects of soil on their transportation to grains. *J. Radiat. Res. Appl. Sci.* **8**, 300–310 (2015).
8. Mostafa, A. M. A., Uosif, M. A. M., Elsaman, Reda, Alrowaili, Z. A. & Moustafa, El-sayed. The dependence of natural radioactivity levels and its radiological hazards on the texture of agricultural soil in Upper Egypt*.* *Environ. Earth Sci.* **79**, 228 (2020). doi:10.1007/s12665-020-08946-z.
9. Hamzah, Z., Rahman, S. A. A. & Saat, A. Measurement of ^226^Ra, ^228^Ra and ^40^K in soil in district of Kuala Krai using gamma spectrometry. *Malays. J. Anal. Sci.* **15**, 159–166 (2011).
10. Almayahi, B, A., Tajuddin, A.A. & Jaafar, M.S. Radiation hazard indices of soil and water samples in Northern Malaysian Peninsula. *Appl. Radiat. Isot*. **70 (11)**, 2652-2660 (2012).
11. Alnassar, N., Jaafar, M. & Kabir, N. Determination of concentrations of natural radionuclides in soils and water in non-cultivated sites in Seberang Perai, Malaysia. *IOSR J. Appl. Phys.* **9**, 27–35 (2017).
12. Priharti, W., Samat S.B., and Yasir, M.S. Measurement of natural radionuclides in Malaysian bottled mineral water and consequent health risk estimation. AIP Conf. Proc. 1678, 040012 (2015).
13. Aswood, M.S., Jaafar, M.S., & Sabar, B. Assessment of radionuclide transfer from soil to vegetables in farms from Cameron Highlands and Penang, Malaysia using neutron activation analysis. *Appl. Phys. Res****.* 5(5),** 85-92 (2013).
14. Priharti W. & Samat S.B. Radiological risk assessment from the intake of vegetables and Fruits in Malaysia*. Malays. J. Analyt. Sci.* **20,** 1247-1253 (2016).
15. Olatunji M.A., Uwatse, O.B., Khandaker, M.U., Amin, Y. & Faruq G. Radiological study on newly developed composite corn advance lines in Malaysia*. Phys. Scri.* **89**, 125002 (2014).
16. Alsaffar, M.S., Jaafar, M.S., Kabir, N.A. & Ahmad, N. Distribution of ^226^Ra, ^232^Th, and ^40^K in rice plant components and physico-chemical effects of soil on their transportation to grains. *J. Radiat. Res. Appl. Sci.* **8**, 300–310 (2015).
17. Abojassim A.A., Hady H.N. & Mohammed Z.B. Natural radioactivity levels in some vegetables and fruits commonly used in Najaf Governorate, Iraq*. J. Bioenergy Food Sci.* **3**, 113-123 (2016).
18. Uosif M.A.M., Alrowaili Z.A., Elsaman Reda. & Mostafa A.M.A. Soil-soybean transfer factor of natural radionuclides in different soil textures and the assessment of committed effective dose. *Radiat. Prot. Dosim*. **88**(4), 529-535, (2020). doi:10.1093/rpd/ncaa005.
